# Spatial regulation of the glycocalyx component podocalyxin is a switch for prometastatic function

**DOI:** 10.1126/sciadv.abq1858

**Published:** 2023-02-03

**Authors:** Alvaro Román-Fernández, Mohammed A. Mansour, Fernanda G. Kugeratski, Jayanthi Anand, Emma Sandilands, Laura Galbraith, Kai Rakovic, Eva C. Freckmann, Erin M. Cumming, Ji Park, Konstantina Nikolatou, Sergio Lilla, Robin Shaw, David Strachan, Susan Mason, Rachana Patel, Lynn McGarry, Archana Katoch, Kirsteen J. Campbell, Colin Nixon, Crispin J. Miller, Hing Y. Leung, John Le Quesne, James C. Norman, Sara Zanivan, Karen Blyth, David M. Bryant

**Affiliations:** ^1^ Institute of Cancer Sciences, University of Glasgow, Glasgow G61 1QH, UK.; ^2^ The CRUK Beatson Institute, Glasgow G61 1BD, UK.; ^3^ Cancer Biology and Therapy Lab, Division of Human Sciences, School of Applied Sciences, London South Bank University, London SE1 0AA, UK.; ^4^ Biochemistry Division, Department of Chemistry, Faculty of Science, Tanta University, Tanta 31527, Egypt.; ^5^ Department of Immunology, The University of Texas MD Anderson Cancer Center, 7455 Fannin Street, Houston, TX 77054, USA.

## Abstract

The glycocalyx component and sialomucin podocalyxin (PODXL) is required for normal tissue development by promoting apical membranes to form between cells, triggering lumen formation. Elevated PODXL expression is also associated with metastasis and poor clinical outcome in multiple tumor types. How PODXL presents this duality in effect remains unknown. We identify an unexpected function of PODXL as a decoy receptor for galectin-3 (GAL3), whereby the PODXL-GAL3 interaction releases GAL3 repression of integrin-based invasion. Differential cortical targeting of PODXL, regulated by ubiquitination, is the molecular mechanism controlling alternate fates. Both PODXL high and low surface levels occur in parallel subpopulations within cancer cells. Orthotopic intraprostatic xenograft of PODXL-manipulated cells or those with different surface levels of PODXL define that this axis controls metastasis in vivo. Clinically, interplay between PODXL-GAL3 stratifies prostate cancer patients with poor outcome. Our studies define the molecular mechanisms and context in which PODXL promotes invasion and metastasis.

## Teaser

Podxl promotes metastasis by acting as a decoy receptor for the invasion-repressing glycocalyx component Galectin-3.

## INTRODUCTION

The cellular glycocalyx is composed of a network of glycoproteins and proteoglycans that coat all cells and that modulates cell interactions with the extracellular matrix (ECM) (*1*). An altered, bulky glycocalyx in cancer cells contributes to tumor progression and metastasis by mechanical regulation of cell-ECM interactions (*2*–*5*). A conundrum in cancer cell glycocalyx constituents influencing ECM interaction is that a number of these are mucins, such as MUC1 or podocalyxin (PODXL), which in normal tissues are not localized to ECM-abutting domains but are instead apically localized and required for apical domain function (*6*, *7*). This suggests a duality in mucin function, promoting normal tissue development at the apical domain or invasion and metastasis when alternately located to ECM-abutting membranes.

The duality of glycocalyx-associated mucins is particularly evident for the sialomucin PODXL. PODXL’s role in promoting apical lumen formation across tissues can be converted to inducing collective apical membrane–driven invasion by interfering with signals that control PODXL removal from ECM-abutting membranes (*7*). This “inverted polarity,” apical proteins localized at the periphery of clusters of epithelial cells rather than at the lumen, is a defining feature of micropapillary cancers that are associated with poor clinical outcome (*8*, *9*). Apical membrane–driven inverted polarity has been proposed as a mechanism for metastasis in colorectal carcinoma (*10*). Elevated PODXL levels are an independent prognostic indicator of disease aggressiveness in several cancers (*11*–*17*). Germline mutations in *PODXL* result in alternate phenotypes; some PODXL mutations are associated with focal segmental glomerulosclerosis and nephrotic syndrome affecting the apical membrane function of PODXL in the kidney (*18*, *19*), whereas other *PODXL* variants are associated with familial prostate cancer aggressiveness (*12*). This highlights that the functional contribution of mucins to the glycocalyx, whether it be to normal tissue function or disruption to this function and induction of metastatic features, must be regulated in a context-specific fashion, such as alternate subcellular targeting to the apical domain or to the ECM-abutting surface.

That PODXL is a transmembrane protein and that its elevated expression is associated with poor clinical outcome in tumors has led to the suggestion of it as a targetable biomarker of metastasis (*20*–*23*). Given that the function of PODXL in normal tissues is also required, it is essential to identify the tumor-specific contexts regulating PODXL function. PODXL expression alone modestly stratifies outcome in some cancers but becomes a potent predictor of poor outcome when comparing cortical to intracellular levels of PODXL (*11*, *23*–*28*). Moreover, tumor-restricted glycosylation patterns have allowed the development of tumor cell–selective therapeutic anti-PODXL antibodies (*29*). This emphasizes that mucins such as PODXL require contextual modulation to form part of the tumor-associated, prometastatic bulky glycocalyx. Here, we determine the molecular mechanisms that control the switch of PODXL toward a prometastatic glycocalyx component. We identify an unexpected function of PODXL as a decoy receptor that relieves the invasion-inhibiting effect of the glycocalyx component galectin-3 (GAL3) and demonstrate that the levels of this switch identify prostate cancer patients with high metastasis and poor outcome.

## RESULTS

### PODXL promotes invasive tunnel formation inside the ECM

**Fig. 1.**
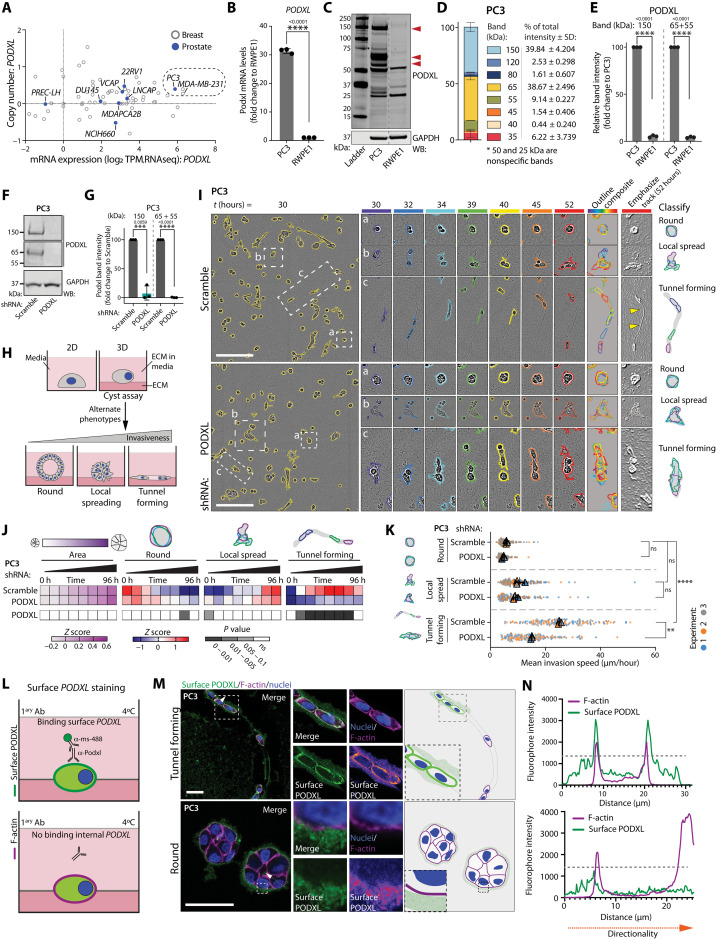
PODXL promotes the formation of invasive tunnels in the ECM. (**A**) *PODXL* mRNA versus copy number. Prostate, blue; breast, gray. Data, CCLE. (**B**) *PODXL* mRNA, RWPE1 versus PC3. Normalized to RWPE1, *n* = 3 independent isolations/reactions. (**C**) Western blot (WB), RWPE1 and PC3 for PODXL and GAPDH (loading control). Arrowheads, major bands. Dashed line, nonadjacent lanes. (**D** and **E**) Relative contribution to total PODXL levels and relative PODXL levels in (C). Major bands, 150 kDa and 55 to 65 kDa; 50- and 25-kDa bands, nonspecific. Normalized to PC3, *n* = 3 independent lysate preparations. (**F** and **G**) Western blot and quantitation, PC3 expressing Scramble or *PODXL* shRNA for PODXL and GAPDH (loading control). Normalized to Scramble, *n* = 3 independent lysate preparations. (**H**) Schema, alternate 3D phenotypes. (**I**) Phase image, PC3 from (F). Cysts, yellow outline. Example of (a) round, (b) local-spreading, and (c) tunnel-forming objects shown. Outlines colored by time in single frames and overlaid. Inverted image, ECM tunnels. Schema, alternate 3D behaviors. (**J**) Heatmap, area, and phenotype classification of (I). *Z*-score–normalized values (blue to red). Heatmap, *P* values (grayscale) compared to Scramble, Student’s *t* test and Cochran-Mantel-Haenszel, Bonferroni-adjusted. *n* = 3 independent experiments, 2 to 3 technical replicates per condition, 254 to 518 cysts per condition per experiment. (**K**) Mean invasion speed (micrometers per hour) of (I). *n* = 3 independent experiments, 38 to 68 cysts per condition per experiment. (**L**) Schema, surface PODXL immunostaining. (**M**) Confocal images and schema, surface PODXL (green or Fire LUT), F-actin (magenta), and nuclei (blue) in tunnel-forming or round 3D PC3. Dotted arrow, described in (N). (**N**) Intensity profiles for surface PODXL (green) and F-actin (magenta) from (M). Dotted line, threshold for background labeling outside cell boundary. Scale bars, (I) 300 μm and (M) 50 μm. *P* values, unpaired Student’s two-tailed *t* test; ns, not significant; **P ≤ 0.005, ***P ≤ 0.0005, and ****P ≤ 0.0001. Bar graphs, means ± SD. Superplots, means ± SD; circles, technical replicates; triangles, average per experiment.

We identified breast and prostate cancer cell lines with high PODXL expression, as PODXL has been associated with invasion and metastasis in these cancers. Nontumorigenic prostate lines PREC-LH (Fig. 1A) (*30*) and RWPE1 cells (Fig. 1B) (*30*) minimally expressed *PODXL* mRNA. In contrast, highly metastatic prostate PC3 (*31*) and breast MDA-MB-231 (*32*) lines displayed the highest *PODXL* mRNA with minimal change to gene copy number (dashed lined square in Fig. 1A and table S1). We therefore explored PODXL function in PC3 and MDA-MB-231 cells.

The predicted molecular weight of PODXL is 58 kDa, but likely due to glycosylation (*33*, *34*), several bands of PODXL were detected in Western blots in both cell lines, most prominently ~150 kDa and 55 to 65 kDa (Fig. 1, C to E, and fig. S1A). Depletion of PODXL via short hairpin RNA (shRNA) (Fig. 1, F and G, and fig. S1, A and B) or single guide RNA (sgRNA)–mediated knockout (fig. S1, C and D) largely abolished both PODXL bands.

We examined the phenotype of PODXL depletion. PODXL-depleted cells were impaired in collective cell invasion specifically when ECM was involved. In scratch-wound assays wherein cell monolayers were plated on and overlaid with ECM, control cells from PC3 and MDA-MB-231 cell lines invaded via leader cells, forming tunnels that were used by follower cells to invade (fig. S1, E to K, yellow arrowheads; and movies S1 and S2), similar to previous observations (*35*, *36*). PODXL-depleted cells failed to form leader cell–induced tunnels in both cell types (fig. S1, F and H), resulting in decreased invasion of the overall population (fig. S1, G and I), suggesting a common motility defect. In contrast to invasion defects in 3D, PODXL-depleted PC3 cells displayed no defect in two-dimensional (2D) migration in scratch-wound migration assays without ECM addition (fig. S1, J to M). No defect was detected in PC3 proliferation in either 2D culture or when embedded into ECM gels to form 3D acini (fig. S1, N and O). PODXL is therefore required for invasion into the ECM.

To determine whether PODXL was essential for tunnel-inducing cells or the movement of follower cells into tunnels, we embedded single PC3 cells into the ECM to induce 3D acinus formation (Fig. 1H). We examined these using wide-field multiday time-lapse imaging, allowing the detection of how clonal outgrowth of a single cell can progress to alternate 3D phenotypes (*37*). By overlaying the outlines of acini over defined time intervals into a single image (outline composite), three phenotypes that occurred in parallel in the control PC3 cells (Scramble, nontargeting shRNA) could be observed: nonmotile, round acini (round); those that only invade locally (local spreading); and those that move back and forward through a seeming conduit in the ECM (tunnel forming) that was reminiscent of leader cells at the front of invasion assays (Fig. 1, I and J; fig. S1F; and movie S3). We emphasized the ECM in phase-contrast images (“emphasize track”; see Materials and Methods), which revealed a tunnel in the ECM.

We developed a method for quantitative assessment of these phenotypes over time (fig. S2A). While round and local-spreading phenotypes could potentially be distinguished by their shape changes at a given static time point, tunnel-forming cells shared many of these shapes but instead moved along conduits in the ECM. By creating outline composites of cells imaged every hour for several days into 12-hour time intervals and converting composites into single objects, we could train a Gentle Fast Boosting machine learning model with high fidelity (92 to 98% accuracy) to user-defined classification to detect the three aforementioned phenotypes. The application of this method to large-scale parallel live imaging of thousands of acini allowed robust statistical analysis of phenotypes, which could be presented in heatmaps that displayed how parameters of interest changed in 12-hour intervals along with statistical analysis across multiple replicates and experiments.

Quantitative assessment revealed that while PODXL-depleted PC3 cells could display modest elongation, they were devoid of the tunnel-forming phenotype, the local-spreading phenotype was unchanged, and switched to a round phenotype modestly upon PODXL knockdown (shRNA; Fig. 1, I and J, and movie S3) or significantly upon PODXL knockout (sgRNA; fig. S2, B and C). Modest effects were observed on the total area (Fig. 1, I and J, and fig. S2, B and C), corroborating a lack of effect of PODXL depletion on proliferation in 3D (fig. S1N). When MDA-MB-231 cells are embedded into the ECM, these initially become highly motile and elongated, also moving back and forward through apparent ECM tracks before developing into a branched multicellular network (fig. S2D and movie S4). Quantitative analysis revealed this as an increase in local-spreading and tunnel-forming activity over time (fig. S2E). Upon PODXL depletion, MDA-MB-231 cells at early time point lost this initial motility and instead switched to a robust local-spreading phenotype (fig. S2, D and E, and movie S4). The analysis of tunnel-forming objects in MDA-MB-231 cells upon PODXL depletion was complicated by the extreme elongation of nonmotile cells in this condition, resulting in a lack of significant reduction in the tunnel-forming phenotype (fig. S2, D and E). Therefore, we directly examined the mean invasion speed of elongating PC3 and MDA-MB-231 cells in 3D culture, rather than using tunnel-forming classification as an indirect proxy for motility. In PC3 acini, PODXL depletion resulted in decreased mean invasion speed specifically in the tunnel-forming population of acini (Fig. 1K and fig. S2F). A reduction in mean invasion speed was also observed in MDA-MB-231 cells upon PODXL depletion (fig. S2G). This suggests that while PODXL depletion can have varying effects on acinus shape, across cell types, there is a consistent reduction of motility in a 3D context. This is further supported by a common reduction of invasion in orthogonal ECM invasion assays in both PC3 and MDA-MB-231 cells (fig. S1, F to I). As this motility in PC3 cells results in the formation of a tunnel, we therefore use tunnel formation in PC3 cells as a method to identify movement over time from static images.

Expression of extracellularly green fluorescent protein (GFP)–tagged PODXL [isoform A; henceforth described as wild type (WT)] in PC3 cells stimulated the tunnel-forming phenotype to occur earlier at the expense of local spreading compared to GFP alone (GFP-PODXL; fig. S3, A to D). Intriguingly, the round phenotype was unaffected by PODXL depletion or overexpression despite stable viral manipulation of both conditions. Labeling of endogenous surface PODXL in nonpermeabilized acini revealed ECM-adjacent labeling in tunnel-forming, but not round, acini (Fig. 1, L to N). In GFP-PODXL–expressing acini, anti-GFP antibody staining to extracellular GFP revealed heterogeneity in surface GFP-PODXL presence despite similar levels of total GFP-PODXL expression (fig. S3E). This suggests that the specificity of PODXL effects on tunnel formation is not due to the lack of PODXL expression in other phenotypes (round and local-spreading) but rather the existence of a specific context that facilitates cortical PODXL localization to promote motility in the tunnel-forming population.

### Differential cortical localization of PODXL controls invasiveness

**Fig. 2.**
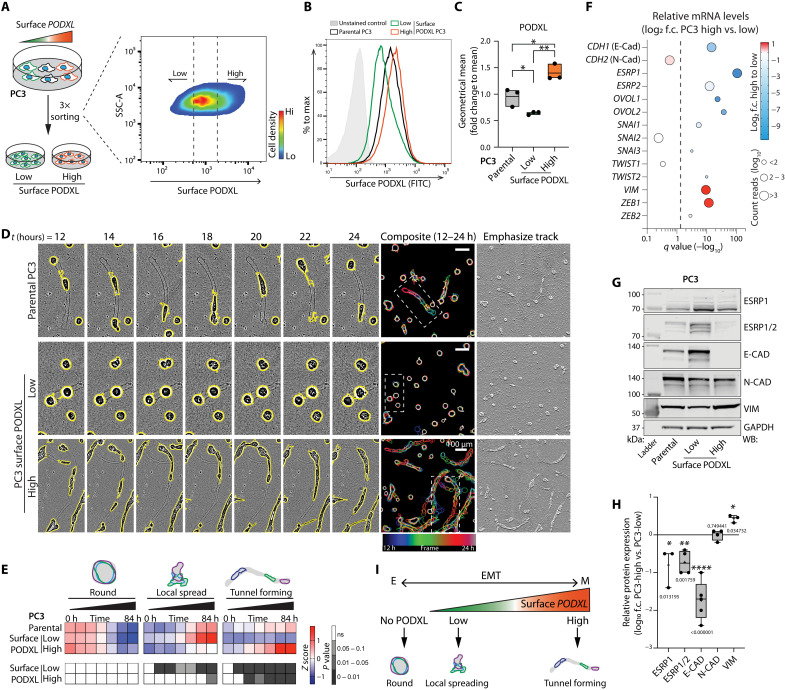
Tunnel-forming invasion is dependent on cortical PODXL. (**A**) Schema, isolation of PC3 subpopulations with low or high surface PODXL levels. (**B** and **C**) Representative plot and geometrical mean from flow cytometry using anti-extracellular PODXL in cells from (A). Normalized to the mean of PC3. Dots, experiments; midline, mean; boundaries, minimum and maximum values; *n* = 3 independent experiments. FITC, fluorescein isothiocyanate. (**D** and **E**) Phase images and quantitation of PC3 from (A). Cysts, yellow outlines. Outlines colored by time in single frames and overlaid. Scale bars, 100 μm. Inverted image, ECM tunnel (emphasize track). Heatmap of relative levels of round, spread, and tunnel-forming PC3, *z*-score normalized. *n* = 4 independent experiments, 3 to 6 technical replicates per condition, 274 to 942 cysts per condition per experiment. (**F**) Bubble plot, mRNA expression of EMT-related genes. *X* axis, *q* value (−log_10_), gray dashed line, significance of *P* = 0.05; color, PC3-high versus PC3-low mRNA expression fold change value (log_2_, blue to red); bubble size, average of normalized count reads of each transcript (log_10_); *n* = 4 independent RNA isolations per condition. (**G**) Western blot and quantitation, PC3 parental or sorted subpopulations for EMT-related markers (ESRP1/2, E-CAD, N-CAD, and VIM) or GAPDH (loading control). High surface versus low surface PODXL levels; intensity fold change (log_10_). Box and whiskers: Dots, replicates; +, mean; midline, median; boundaries, quartiles; *n* = 3 to 5 independent experimental isolations of cell lysates. (**I**) Schema. Relationship between PODXL, EMT, and phenotype. (C) *P* values, unpaired Student’s two-tailed *t* test; (**H**) *P* values, multiple unpaired *t* test, Welch’s correction. *P ≤ 0.05, **P ≤ 0.005, and ****P ≤ 0.0001. Heatmap, *P* values, Cochran-Mantel-Haenszel and Bonferroni-adjusted, compared to control, grayscale as indicated.

We examined the context in which PODXL can promote invasive tunnel formation and whether this was related to cortical presentation of PODXL. Three rounds of serial sorting for endogenous surface PODXL levels allowed the recovery of cell populations with surface PODXL at high or low levels (Fig. 2, A to C). Notably, cells with altered surface levels of PODXL displayed different frequencies of local-spreading and tunnel-forming 3D phenotypes; low–surface PODXL cells lost the ability to form tunnels, while high–surface PODXL cells were highly tunnel-forming (Fig. 2, D and E, and movie S5). Consequently, high–surface PODXL cells formed elongated tunnels that allowed rapid invasion of the cell population in ECM invasion assays (fig. S4A, yellow arrowheads, and movie S6), while wounded monolayers of low–surface PODXL cells lacked invasive chains and were poorly invasive (fig. S4, A to C). No proliferation differences were detected between high– and low–surface PODXL–expressing cells in either 2D or 3D (fig. S4, D and E), similar to the effects of PODXL depletion on parental cells (fig. S1, N and O).

The contrast in surface levels between high– and low–surface PODXL cells was not reflected in major mRNA or total protein level changes of either PODXL or its known major binding partners (Ezrin, *SLC9A3R1*/NHERF1, and *SLC9A3R2*/NHERF2); only a small reduction in *EZRIN* mRNA, but not protein, occurred (fig. S5, A to C), while total PODXL protein levels showed a modest difference between high-surface and low-surface stable lines (1.68-fold change; fig. S5, B and C). Robust overexpression of GFP-PODXL (fig. S4, F and G) in low-surface stable lines did not increase tunnel formation (fig. S4H), similar to a lack of effect in the round population in GFP-PODXL–overexpressing parental PC3 cells (fig. S3, A to D). This supports the notion of a cellular context that promotes surface PODXL and invasion rather than a difference in PODXL complex expression between high– and low–surface PODXL cells.

Analysis of RNA sequencing (RNA-seq) of parental, high–, and low–surface PODXL lines revealed significant enrichment of pathways associated with epithelial-mesenchymal transition (EMT) between sublines (fig. S5D). Low–surface PODXL cells presented an epithelial profile with enrichment of epithelial transcripts (*CDH1* and *ESRP1/2*), whereas high–surface PODXL cells presented a mesenchymal profile (*VIM* and *ZEB1*; Fig. 2F), data confirmed at the protein level (Fig. 2, G and H). This trend was maintained in both 2D and 3D cultures (fig. S5, E and F). This suggested that an EMT provides the context that promotes increased cortical levels of PODXL, independent of regulating the total levels of PODXL or major known PODXL-binding partners. This raised the possibility that cortical PODXL might simply be a functionally irrelevant passenger event that nonetheless could be used to select for a mesenchymal mode of invasion. However, depletion of PODXL in high–surface PODXL cells attenuated the ability of these to form tunnels and the entire population of cells to invade (fig. S5, G to K). This confirms a functional role of PODXL in mesenchymal-type invasion.

Despite reports that PODXL expression is increased and required for full EMT effects (*38*, *39*), we saw variable contribution of PODXL to EMT status. PODXL overexpression in low-surface PODXL PC3 cells increased epithelial characteristics (E-cadherin levels; fig. S4, F and G), while PODXL depletion in PC3 high–surface PODXL cells also increased E-cadherin levels (fig. S5, L and M). In contrast, in MDA-MB-231 cells, PODXL depletion increased epithelial and decreased mesenchymal markers (fig. S5, N and O). This reveals a complex contribution of PODXL to the EMT status of cells, which differs in different contexts. In PC3 cells, invasion is related to increased cortical levels of PODXL, not only the EMT status of cells (Fig. 2I).

### Ubiquitination controls cortical levels of PODXL

**Fig. 3.**
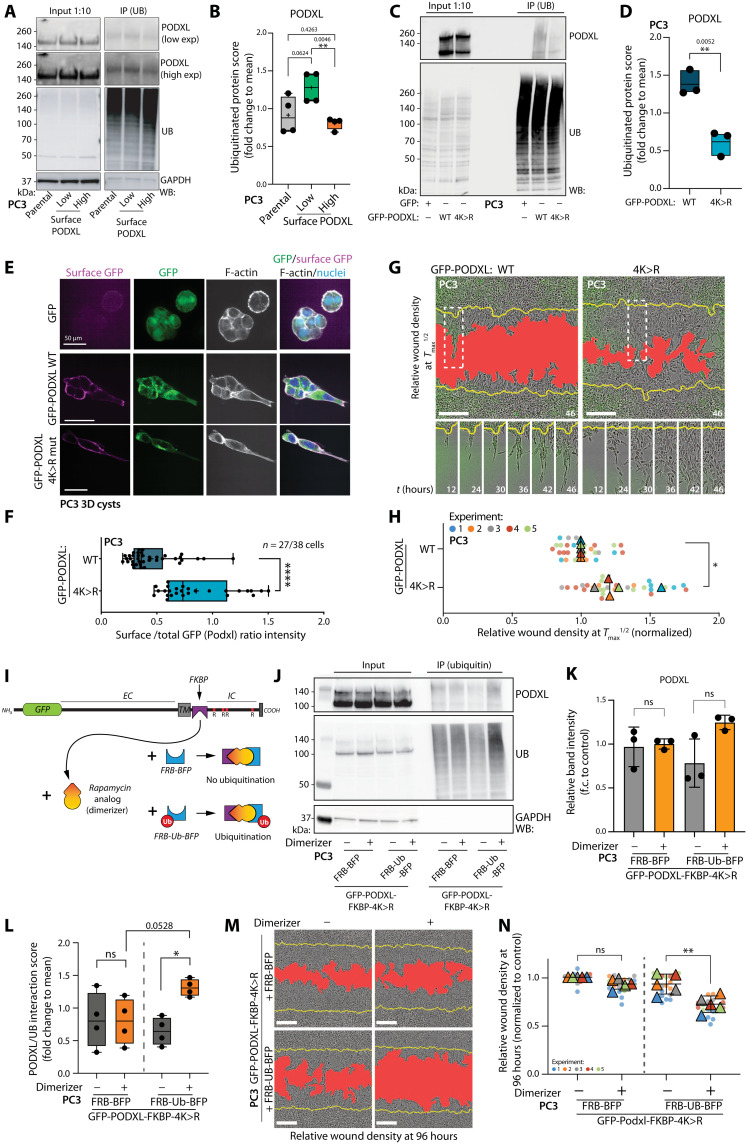
PODXL ubiquitination controls cortical levels. (**A** to **D**) Western blot and quantitation, in PC3 and (A and B) subpopulations sorted for PODXL surface levels or (C and D) expressing GFP or GFP-PODXL WT or 4K>R mutant for PODXL, ubiquitin, and GAPDH (loading control). Box and whiskers: Dots, replicates; +, mean; midline, median; boundaries, quartiles; (B) *n* = 4 independent lysate preparations and protein isolations; (D) *n* = 3 independent experiments. (**E**) Immunofluorescence in PC3 cysts expressing GFP, GFP-PODXL WT, or GFP-PODXL 4K>R using anti-GFP antibody. Surface GFP (magenta), total GFP (green), F-actin (gray), and nuclei (blue). (**F**) Quantitation of surface:total GFP (PODXL) intensity ratio from (E). Box and whiskers: Dots, replicates; +, mean; midline, median; boundaries, quartiles; *n* = 1 and 2 technical replicates, 27 to 38 cysts analyzed per condition. (**G** and **H**) Phase images and quantitation. Yellow line, initial wound; red, wound at *T*_max_^1/2^ invasion. Circles, technical replicates (*n* = 4 to 8 per experiment); triangles, average per independent experiment (*n* = 5); color, experiment number. Means ± SD. (**I**) Cartoon, inducible ubiquitination of PODXL. (**J** to **L**) Western blotting and quantitation, GFP-PODXL (FKBP + 4K>R) with or without dimerization and coexpression of FRB-BFP or FRB-ubiquitin-BFP for PODXL, ubiquitin, and GAPDH (loading control). Quantitation of total levels of PODXL, normalized to average, *n* = 4 independent experiments. (**M** and **N**) 3D invasion of (J), phase images and quantitation as described in (G). Circles, technical replicates (*n* = 2 to 5 per experiment); triangles, average per independent experiment (*n* = 5); color, experiment number. Means ± SD. Scale bars, (E) 50 μm and (G and M) 300 μm. *P* values, unpaired Student’s two-tailed *t* test, *P ≤ 0.05, **P ≤ 0.005, and ****P ≤ 0.0001.

As cell populations sorted for different PODXL surface levels had similar PODXL mRNA and total protein levels, we focused on ubiquitination as a potential mechanism to control PODXL abundance at the cell cortex (*40*). Endogenous PODXL ubiquitination was significantly altered between cells with different surface levels of PODXL; cells with low surface PODXL levels show strong ubiquitination of PODXL (Fig. 3, A and B). Publicly available datasets indicated that ubiquitination occurs on all four cytoplasmic lysine residues of PODXL (K431/525/526/547; phosphosite.org). Mutation of these four residues in GFP-PODXL (K431/525/526/547R; 4K>R; fig. S6A) resulted in a strong attenuation of ubiquitination (Fig. 3, C and D). Ubiquitination-deficient PODXL (GFP-PODXL 4K>R) cortical levels were enhanced (Fig. 3, E and F, and fig. S6, B and C) and invasive capacity increased (Fig. 3, G and H) compared to WT PODXL (GFP-PODXL WT). This reveals that low levels or a lack of PODXL ubiquitination is associated with an EMT, which facilitates high cortical expression of PODXL. Notably, ubiquitination-deficient PODXL (GFP-PODXL 4K>R) was able to drive invasion in the absence of an EMT (fig. S6, D to G), suggesting that the ubiquitination status of PODXL is a major target of EMT processes, and PODXL is an amplifier of mesenchymal invasion, not a passenger molecule.

To determine, conversely, whether ubiquitination drives low surface levels of PODXL, we created an experimentally inducible PODXL ubiquitination system based on rapalog-inducedFK506 binding protein (FKBP) and FKBP–rapamycin binding (FRB) domains oligomerization (Fig. 3I) (*41*). We inserted an FKBP dimerization domain into the cytoplasmic domain of nonubiquitinatable 4K>R mutant PODXL immediately after the transmembrane domain, followed by the cytoplasmic tail. We coexpressed a TagBFP-tagged FRB dimerization domain fused to ubiquitin (FRB-Ub-BFP) or a dimerization domain lacking ubiquitin as a control (FRB-BFP). Only the combination of FRB-Ub-BFP and dimerizer robustly induced PODXL ubiquitination (Fig. 3, J to L), decreased the surface level of PODXL (fig. S6, H and I), and reduced the invasive ability of the cell population (Fig. 3, M and N). These manipulations altered PODXL localization without change in total GFP-PODXL expression (Fig. 3, J and K). These data indicate that differential ubiquitination of PODXL is the key context provided by an EMT controlling whether PODXL participates in invasive activities.

### GAL3 interacts with the extracellular domain of N-glycosylated PODXL

**Fig. 4.**
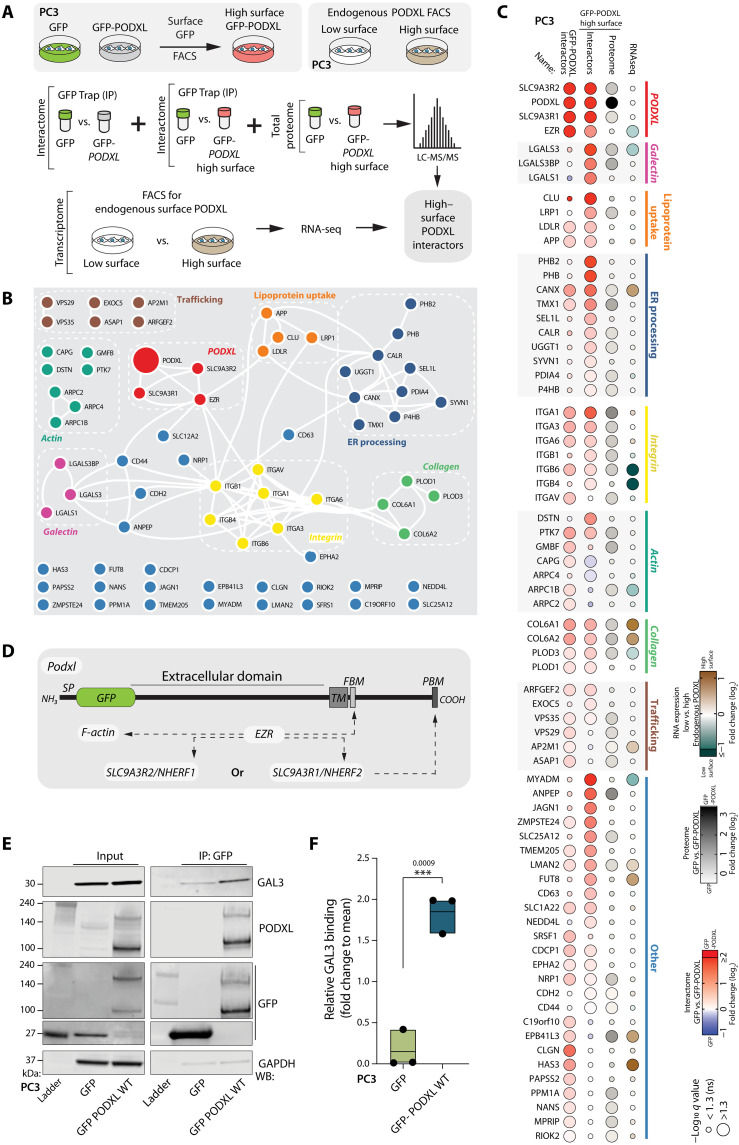
Multiomic interrogation reveals GAL3 as a cortical PODXL interactor. (**A**) Schema, multiomic (interactome, proteome, and transcriptome) profiling to identify high–surface PODXL interactors. (**B**) Network diagram of subgroup interactions among identified PODXL-binding partners. Networks, STRING analysis; layout, Cytoscape analysis. (**C**) Bubble plot, PODXL-binding partners sorted by subgroup then interaction score with high surface PODXL. For proteomics, color represents fold change (log_2_) interaction with PODXL compared to GFP alone; interactome, blue to red; proteome, grayscale. For RNA-seq, color represents fold change (log_2_) endogenous high– compared to low–surface PODXL expression. Bubble size, significance (−log_10_). (**D**) Cartoon, GFP-PODXL domains and known PODXL interactors. SP, signal peptide; TM, transmembrane domain; FBM, FERM-binding motif; PBM, PDZ-binding motif. (**E**) GFP-Trap immunoprecipitation of GFP or GFP-PODXL WT from PC3 cells. Western blotting of input and immunoprecipitates for GAL3, PODXL, GFP, and GAPDH (loading control for GFP). (**F**) Quantitation of relative GAL3 to precipitated GFP versus GFP-PODXL WT in PC3 cells. Floating bar graph: dots, experiments; midline, mean; *n* = 3 independent experimental culturing, lysate preparation, and protein isolation. *P* values (unpaired Student’s two-tailed *t* test), ***P ≤ 0.0005.

That ECM-adjacent location of PODXL was essential for its function prompted us to investigate cortical PODXL interactors. We used mass spectrometry (MS)–based proteomics to compare the interactome of PC3 cells overexpressing either GFP, GFP-PODXL, or GFP-PODXL sorted for high surface levels, isolated by GFP-Trap precipitation (Fig. 4A). We used stable isotope labeling with amino acids in cell culture (SILAC) for accurate protein quantification. We cross-referenced this with the total proteome of GFP-PODXL high surface cells and with RNA-seq comparing PC3 subpopulations sorted for endogenous PODXL at high surface versus low surface levels (Fig. 4A).

We identified multiple interactors of PODXL, many of which could be organized into functional complexes using STRING network analysis (Fig. 4, B and C). In addition to the reported core PODXL complex (PODXL, SLC9A3R1, SLC9A3R1, and EZR) (Fig. 4D) (*7*, *42*–*44*), this included not only networks for glycocalyx-associated components including galectins, integrins, and collagen but also lipoprotein uptake, endoplasmic reticulum processing, actin regulation, trafficking, and additional proteins not easily assigned to a complex (Fig. 4, B and C). The core PODXL complex was the strongest interaction group, but notably, these interactions were unchanged between GFP-PODXL and GFP-PODXL sorted for high expression (Fig. 4C, top clustered group). This excludes changes in the core complex underpinning differential cortical localization.

We noted robust induction of association of glycocalyx-associated galectins with high surface GFP-PODXL, while these were low in GFP-PODXL alone (Fig. 4, B and C). LGALS1/GAL1 and LGALS3/GAL3 are β-galactoside–binding lectins and components of the tumor-associated bulky glycocalyx that regulate the function and structure of glycoproteins (*45*). GAL1 has been shown to promote the prometastatic function of the glycocalyx (*3*), whereas GAL3 has been reported as a promoter of several kinds of cancer; data from prostate cancer are controversial (*46*); GAL3 is higher in benign prostatic hyperplasia and lower in adenocarcinoma compared to control patients (*46*, *47*). Of this galectin interaction group, GAL3 was the strongest interactor of high surface GFP-PODXL (Fig. 4C). We confirmed the association of PODXL with GAL3 in independent immunoprecipitations (Fig. 4, E and F, and fig. S7, A to J), including that nonubiquitinatable PODXL (GFP-PODXL 4K>R), which has higher surface presentation, showed increased association with GAL3 compared to WT PODXL (fig. S7, D and E).

GAL3 exists in both extracellular and intracellular pools. We mapped the interaction site of GAL3 on PODXL (fig. S7, A to G). GAL3 association with PODXL was maintained with both isoforms of PODXL (PODXLa and PODXLb), upon mutation of binding sites for Ezrin (Ferm-binding motif; FBM*) or NHERF1/2 (ΔPDZ motif) either alone or together, or upon fusion of Ezrin in place of the PDZ motif (ΔPDZ + Ezr). This suggests that GAL3 association is independent from core complex association. Deletion of the entire PODXL cytoplasmic domain (GFP-PODXL ΔIC) still resulted in robust association of GAL3 with PODXL (fig. S7, F and G), revealing that the association is extracellular. We were unable to recover stable expressers of PODXL with the extracellular domain deleted (GFP-PODXL ΔEC). In contrast to the lack of effect of the mutants above, mutation of N-linked glycosylation sites in PODXL [5N>Q mutant; (*48*)] abolished GAL3 interaction (fig. S7, A to C). Reciprocally, mutation of the GAL3 carbohydrate recognition binding domain (CRD) to uncouple the association of N-acetyllactosamine carbohydrate glycoprotein motifs (R186S; fig. S5H) (*49*) abolished the association of GAL3 with PODXL (fig. S7, I and J). Although we cannot rule out indirect association due to the potential effects of these mutations on other interaction partners, these data suggest that GAL3 associates with the N-glycosylated PODXL extracellular domain via the GAL3 CRD.

### GAL3 controls invasion by regulating surface levels of PODXL

PODXL manipulations that increased surface levels (i.e., GFP-PODXL sorted for high levels, 4K>R) also increased tunnel-forming activity and invasion (Fig. 3, G and H, and fig. S7, K to M), as well as increased GAL3 association (Fig. 4C and fig. S7, D to G). This initially suggested that GAL3 might be required for the cortical function of PODXL. However, this contrasted with *LGALS3* (GAL3) mRNA expression being the highest in the noninvasive PC3-low subpopulation and lowered in the invasive high-surface cells (fig. S7N). Extracellular levels of GAL3 protein were also robustly increased in low–surface PODXL cells (fig. S7, O and P). This suggests that extracellular GAL3 might repress PODXL function. We investigated whether GAL3 was a positive or negative invasion regulator.

**Fig. 5.**
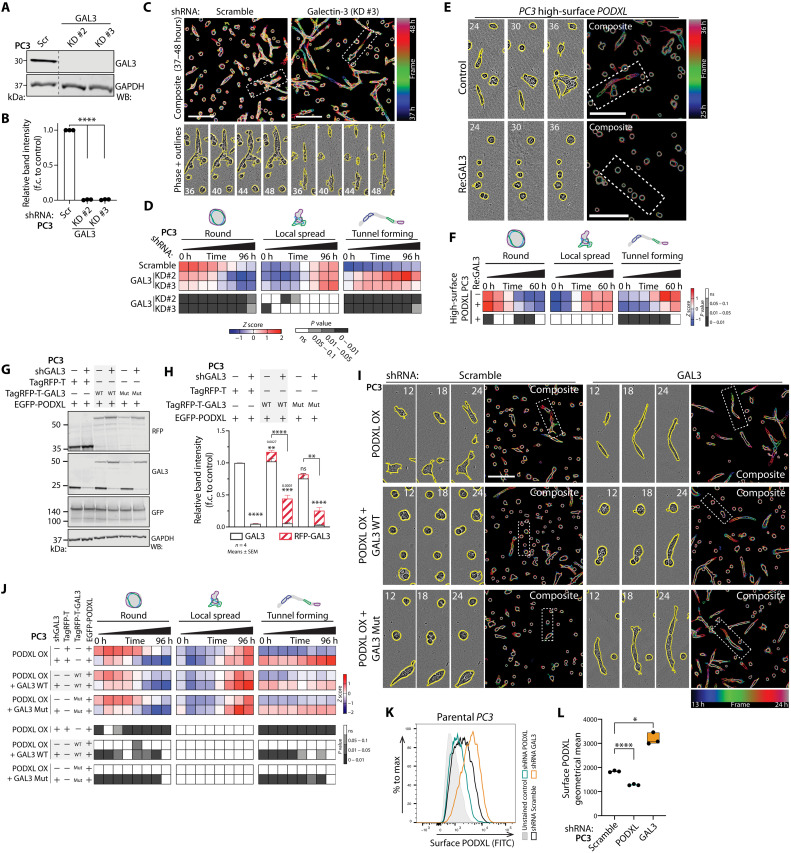
GAL3-PODXL is a mutually antagonistic ligand-receptor pair. (**A** and **B**) Western blot and quantitation, PC3-high expressing Scramble or *GAL3* shRNAs for GAL3 or GAPDH (loading control). Lines, not adjacent in gel. Normalized to Scramble, means ± SD, *n* = 3 independent experiments. (**C** and **D**) Phase images and quantitation of (A). Cysts, yellow outlines. Outlines colored by time in single frames and overlaid. Heatmap, proportions of round, spread, and tunnel forming, *z*-score–normalized. *n* = 3 independent experiments, 4 technical replicates per condition, 1063 to 1789 cysts per condition per experiment. (**E** and **F**) Phase images and quantitation of PC3-high cysts (control) with Re:GAL3. Cysts, yellow outlines. Outlines colored by time in single frames and overlaid. Heatmap, proportions of round, spread, and tunnel forming, *z*-score–normalized. *n* = 3 independent experiments, 3 technical replicates per condition, 297 to 577 cysts per condition per experiment. (**G** and **H**) Western blot and quantitation, PC3 overexpressing GFP-PODXL WT, coexpressing either TagRFP-T, TagRFP-T-GAL3 (WT or R186S mutant), and Scramble or *GAL3* shRNA for RFP, GAL3, GFP, or GAPDH (loading control for RFP). Combined band intensity of GAL3 relative to control. Means ± SD, *n* = 4 independent experiments. (**I** and **J**) Phase images and quantitation of cysts from (G). Cysts, yellow outlines. Outlines colored by time in single frames and overlaid. Heatmap, proportions of round, spread, and tunnel forming, *z*-score normalized. *n* = 2 independent experiments, 3 technical replicates per condition, 287 to 551 cysts per condition per experiment. (**K** and **L**) Representative plot and geometrical mean of surface PODXL levels in Scramble, *PODXL*, or *GAL3* shRNA cells. Bar chart: Dots, experiments; midline, mean; boundaries, minimum and maximum values; *n* = 3 independent experiments. Heatmap *P* values, Cochran-Mantel-Haenszel test, Bonferroni-adjusted, comparing proportion to control, grayscale as indicated. Scale bars, 300 μm. *P* values, unpaired Student’s two-tailed *t* test; *P ≤ 0.05, **P ≤ 0.005, ***P ≤ 0.0005, and ****P ≤ 0.0001.

GAL3 depletion induced extreme tunnel-forming activity (Fig. 5, A to D). Addition of recombinant extracellular GAL3 [recombinant GAL3 (reGAL3)] to the ECM strongly suppressed tunnel formation in high–surface PODXL cells (Fig. 5, E and F). As expected from the heterogeneous invasion context of parental cells, overexpression of GFP-PODXL alone failed to induce tunnel formation across most 3D acini (Fig. 5, G to J). However, combining this with GAL3 depletion resulted in most 3D structures becoming tunnel-forming. Restoration of GAL3 WT expression [RNA interference (RNAi)–resistant TagRFP-T-GAL3], but not non–PODXL-binding GAL3 mutant (R186S), repressed tunnel-forming activity at late time points (Fig. 5, G to J; note the lack of significance in tunnel formation from 60 to 96 hours). This was related to PODXL surface levels as GAL3 depletion increased surface PODXL levels in parental PC3 cells and low–surface PODXL cells but not high–surface PODXL cells that already expressed lowered levels of GAL3 (Fig. 5, K and L, and figs. S7N and S8, A and B). Similarly, stimulation with reGAL3 was sufficient to decrease the surface levels of PODXL both in parental and high–surface PODXL–expressing cells (fig. S8, C to E). This suggests that GAL3-PODXL is a mutually antagonistic ligand-receptor pair, wherein GAL3 triggers internalization of PODXL from the surface, but in doing so, PODXL reduces extracellular GAL3 levels. Accordingly, depletion of PODXL resulted in an increase specifically in the extracellular pool of endogenous GAL3 (fig. S8, F and G). In cells depleted for endogenous GAL3, the addition of reGAL3 resulted in its rapid internalization but not when PODXL was codepleted (fig. S8, H to J). Therefore, GAL3-PODXL is a ligand-receptor pair that triggers cointernalization of both molecules.

### GAL3-PODXL binding prevents GAL3 repression of integrin-dependent invasion

Experimental manipulations that removed the ability of PODXL to be ubiquitinated (4K>R; Fig. 3D) enhanced the association of GAL3 with PODXL (fig. S7, D to G), yet these cells were uncoupled from the inhibitory effect of GAL3 on invasion (Fig. 3, G and H, and fig. S7, K to M). Conversely, expression of a non–GAL3-binding PODXL mutant (N-linked glycosylation deficient; 5N>Q) uncoupled GAL3 association (fig. S7, A to C) but was unable to restore invasion and tunnel-forming activity to PODXL-depleted cells, as could be seen when knockdown cells were rescued with WT PODXL (fig. S7Q). This contrasts with depletion of GAL3 strongly inducing tunnel-forming activity (Fig. 5, A to D). This suggests that GAL3 is inhibitory to invasion, and this inhibition is relieved by binding to PODXL. Therefore, PODXL may be a decoy receptor to relieve a repressive extracellular function of GAL3; uncoupling GAL3 from PODXL does not relieve the repression that GAL3 confers on other invasion-regulating pathways.

Our PODXL interactome analysis revealed the association of PODXL with cell-ECM interactors (integrins and collagen) (Fig. 4, B and C), particularly the enhanced association of β1-integrin with high surface level of PODXL. A previous report indicated that PODXL repressed surface levels of β1-integrin in ovarian cancer cells (*23*). GAL3 associates with β1-integrin (*50*) and controls its surface distribution and clathrin-independent β1-integrin internalization (*51*, *52*). We speculated that GAL3-PODXL cointernalization might act to relieve GAL3-dependent integrin internalization, therefore promoting integrin function in invasion.

We used flow cytometry to measure steady-state surface levels of total and active integrins. Despite equivalent total β1-integrin levels between parental and sublines (fig. S9A), low–surface PODXL cells display a strong reduction in the ratio of active:total β1-integrin steady-state surface levels, whereas high–surface PODXL cells have robust levels of cortical active:total β1-integrin ratio (fig. S9, B to F). Accordingly, overexpression of GFP-PODXL WT or 4KR mutant increased the active:total surface ratio of β1-integrin (fig. S9, G to L). PODXL depletion decreased the surface levels of active β1-integrin (*P* = 0.0845; fig. S9, M to Q). In contrast, GAL3 depletion did not affect the active:total surface β1-integrin ratio but rather increased the absolute levels of β1-integrin on the cell surface and consequently the absolute levels of active surface β1-integrin (fig. S9, M to P). This reveals that surface PODXL is associated with β1-integrin activation on the cell surface, while GAL3 controls total β1-integrin cell surface levels.

**Fig. 6.**
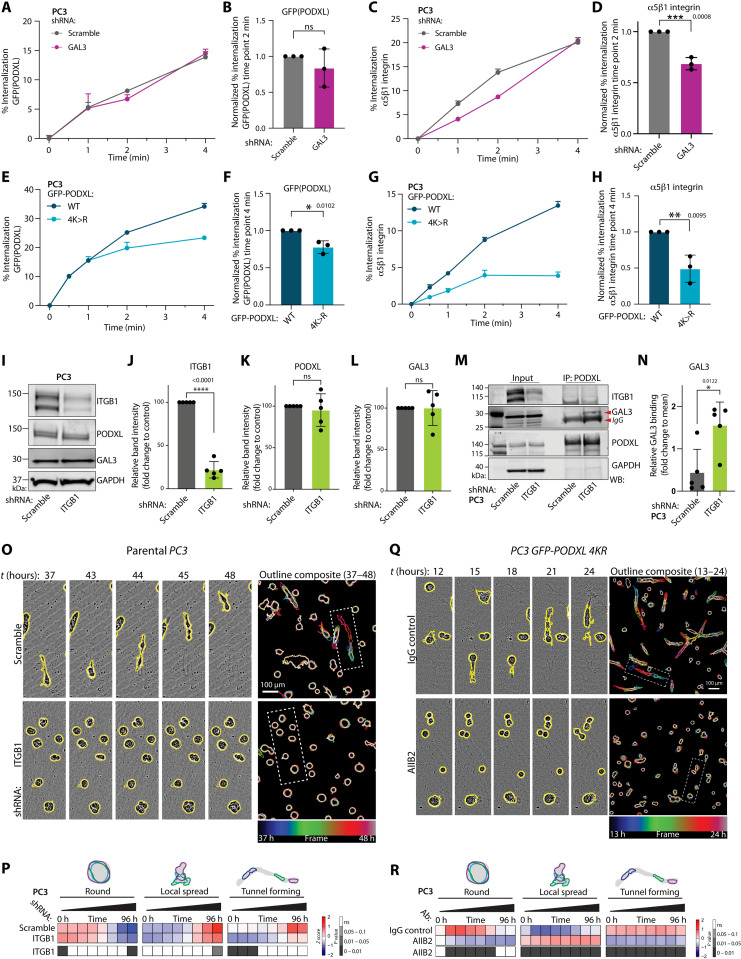
PODXL is a decoy receptor for GAL3 to release GAL3 repression of integrin-dependent invasion. (**A** to **H**) Receptor internalization by capture ELISA from GFP-PODXL WT–expressing PC3 cells coexpressing Scramble or GAL3 shRNA (A to D) or in cells with GFP-PODXL WT versus 4K>R mutant expression (E to H). Representative graphs, percentage of GFP-PODXL (A), a5β1-integrin (C), and GFP-PODXL WT versus 4K>R (E and G) internalization at indicated time points. Values, means ± SEM, *n* = 3 independent experiments. Graphs showing relative endocytosis at time point of 2 min (B and D) or 4 min (F and H) normalized to control; three independent experiments combined. (**I** to **L**) Western blotting and quantitation, PC3 expressing Scramble or *ITGB1* shRNA for ITGB1, PODXL, GAL3, and GAPDH (loading control). Quantitation across replicates. Means ± SD, *n* = 5 independent experiments. (**M** and **N**) Co-IP, Western blotting, and quantitation of endogenous PODXL and GAL3 from PC3 in (I) for ITGB1, GAL3, PODXL, and GAPDH (loading control). Quantitation across replicates. Means ± SD, *n* = 5 independent experiments. (**O** to **R**) Phase images and quantitation from cells expressing (O and P) Scramble or *GAL3* shRNAs or (Q and R) GFP-PODXL 4K>R mutant (IgG control) with β1-integrin blockade (AIIB2, blocking antibody). Cysts, yellow outlines. Outlines colored by time in single frames and overlaid. Heatmap, proportions of round, spread, and tunnel forming, *z*-score normalized. (P) *n* = 4 independent experiments, 3 technical replicates per condition, 444 to 702 cysts per condition per experiment. (R) *n* = 3 independent experiments, 2 technical replicates per condition, 137 to 354 cysts per condition per experiment. Bar graphs, *P* values, unpaired Student’s two-tailed *t* test; *P ≤ 0.05, **P ≤ 0.005, ***P ≤ 0.0005, and ****P ≤ 0.0001. Heatmap, *P* values, Cochran-Mantel-Haenszel test Bonferroni-adjusted, to compare the area and proportion of each classification to control, grayscale as indicated. Scale bars, 100 μm.

We used a quantitative biochemical approach [capture enzyme-linked immunosorbent assay (ELISA)] to directly measure whether altered surface levels of integrin may be related to its internalization (*53*). Whereas GFP-PODXL WT internalization was not altered in GAL3-depleted versus control cells at the time points measured, GAL3-depleted cells presented a significant decrease in a5β1-integrin internalization (Fig. 6, A to D). Ubiquitin-deficient PODXL (GFP-PODXL 4K>R), which has increased association with GAL3 (fig. S7, D and E), exacerbated this effect, displaying slowed internalization of PODXL (Fig. 6, E and F) and robust inhibition of a5β1-integrin internalization (Fig. 6, G and H). This suggests that GAL3-PODXL interaction may control invasion by regulating integrin surface levels and association with the ECM. Accordingly, cells sorted for endogenous high–surface PODXL levels or expressing GFP-PODXL (WT or 4K>R) showed enhanced association with Matrigel and collagen I but not fibronectin or laminin-1 (fig. S10, A to D). Moreover, while cells depleted for PODXL and expressing control GFP alone formed round acini surrounded by collagen IV, rescue with RNAi-resistant GFP-PODXL WT or 4K>R expression resulted in cells forming tunnels lined by collagen IV (fig. S10E and movie S7). As this collagen IV antibody detects mouse collagen IV provided by the Matrigel, this reveals that one function of ECM-adjacent PODXL is to promote the organization of the ECM to form collagen-lined invasion tunnels.

We tested whether β1-integrin was a key effector of surface PODXL–driven matrix alteration to form ECM tunnels. ShRNA-mediated stable depletion of β1-integrin (ITGB1) did not affect the expression of PODXL or GAL3 (Fig. 6, I to L) but rather enhanced the GAL3-PODXL interaction (Fig. 6, M and N). Depletion of endogenous β1-integrin or inhibition with a function-blocking antibody (AIIB2) abolished tunnel-forming ability in acini (Fig. 6, O and P, and fig. S10, F and G). Crucially, the high-level tunnel-forming activity of nonubiquitinatable PODXL (GFP-PODXL 4K>R) was completely abolished by treatment with β1-integrin function-blocking antibody (AIIB2; Fig. 6, Q and R). Collectively, these data demonstrate that (i) GAL3-PODXL is a ligand-receptor pair whose (ii) cointernalization acts as a decoy for GAL3, thereby (iii) relieving GAL3-directed integrin internalization and (iv) resulting in integrin-dependent remodeling of collagen in the ECM to form invasive tunnels.

### PODXL cortical localization promotes metastasis in vivo

**Fig. 7.**
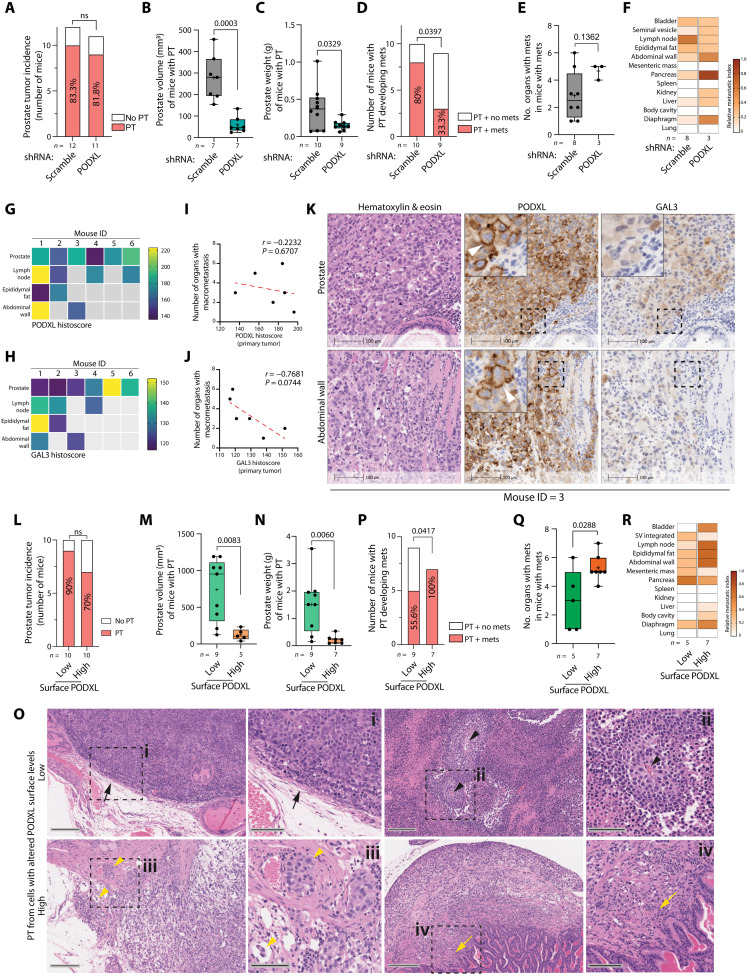
PODXL cortical levels regulate metastasis in vivo. (**A** to **R**) PT and metastasis in mice injected with PC3 (A to K) expressing Scramble or *PODXL* shRNA, *n* = 12 mice per condition or (L to R) sorted for low or high surface PODXL, *n* = 10 mice per condition. (A and L) PT incidence. In mice with PT, (B and M) prostate volume by ultrasound, (C and N) prostate weight, (D and P) the number of mice with metastases, (E and Q) and the number of metastasis-containing organs per mouse; in mice with metastasis, (F and R) metastasis site tropism. (G and H) Heatmap, weighted histoscore for PODXL or GAL3 across six control mice (Scramble) and for PT (prostate) or metastatic sites. (I and J) Pearson correlation of histoscore from PT of six control mice (Scramble) with the number of organs bearing macrometastasis. (K) Serial sections from mouse ID = 3 of the PT (prostate) and metastasis (abdominal wall), stained for H&E, PODXL, or GAL3. Scale bars, 100 μm. Arrowhead, cortical PODXL. (O) Images at intermediate magnification (H&E; scale bars, 200 μm) with high-magnification inserts (H&E; scale bars, 100 μm). Low–surface PODXL tumors (i and ii), black arrows, solid growth with rounded contours; black arrowheads, geographic necrosis with sparse islands of viable tumor around capillaries. High–surface PODXL tumors (iii and iv), yellow arrowheads, looser architecture, infiltrative growth with frequent lymphovascular space invasion; yellow arrow, infiltrative focus. Box-and-whiskers plots, dots, values from individual mice; +, mean; midline, median; boundaries, quartiles; error bars, minimum to maximum values; *n* = 3 to 10 mice that developed PT. *P* values (chi-square test on raw counts for contingency tables or unpaired Student’s two-tailed *t* test for box-and-whiskers plots..

To assess the in vivo function of PODXL in tumorigenesis, we performed orthotopic intraprostatic xenografts of PC3 cells manipulated for PODXL expression or localization into CD1-nude male mice (fig. S11A). At the 8-week end point, this allows for the assessment of primary tumor (PT) formation and multiorgan metastasis (*37*). PODXL-depleted cells showed no defect in engraftment (PT; Fig. 7A). Before sacrifice at 8 weeks after transplantation, ultrasound imaging revealed smaller prostate tumors under the PODXL-depleted condition compared to control shRNA-expressing cells (both with seven mice per condition; Fig. 7B). Macroscopic observation confirmed tumors in additional mice, resulting in 10 control and 9 PODXL-depleted PTs, again confirming smaller tumors via decreased prostate weight under the PODXL-depleted condition (Fig. 7C). In mice with a PT, PODXL-depleted cells showed a 2.4-fold reduction in the number of organs that contained macrometastases (detected in 8 of 10 mice, control; 3 of 9 mice, PODXL depleted; *P* = 0.0397 using chi-square analysis on raw values; Fig. 7D). In the low number of mice that still managed to form metastases from PODXL-depleted cells (*n* = 3, PODXL depleted), these showed no apparent difference in the number of organs with macrometastases per mouse or the tissue tropism of metastasis (Fig. 7, E and F). This suggests that a major role in vivo of PODXL is in tumor formation and metastasis rather than tissue tropism.

Using a weighted histoscore for intensity that accounts for regional differences within a tumor, both PODXL and GAL3 showed no clear pattern or directionality of change in total labeling between primary prostate tumors and metastases (*n* = 6 shRNA scramble control mice with PTs and metastasis; Fig. 7, G and H). However, total GAL3, but not PODXL, levels in the prostate tumor showed an inverse association with the number of organs displaying macrometastases (*r* = −0.7681, *P* = 0.0744, *n* = 6 mice; Fig. 7, I and J). Mirroring the heterogeneity in surface levels of PODXL in PC3 cells in vitro, control PC3 prostate tumors showed variable PODXL localization, with regions of clear cortical PODXL labeling (Fig. 7K, arrowheads, and fig. S11B) and regions of positive staining lacking cortical PODXL. In serial sections of these tumors, GAL3 labeling also displayed regional differences in labeling, which was not mutually exclusive to regions of high surface PODXL. In contrast, examination of metastases to sites common to all mice with metastases (lymph node, epididymal fat, and abdominal wall) revealed prominent cortical PODXL localization (arrowheads) in most metastases (Fig. 7K and fig. S11B, arrowheads) and low but not absent levels of GAL3 in most metastases. Collectively, this suggests an association of high surface localization of PODXL with metastasis.

To test this association, we performed intraprostatic transplantation of PC3 cells sorted for alternate surface PODXL levels (low surface versus high surface) (fig. S11A). These two conditions similarly showed no difference in engraftment (Fig. 7L). Ultrasound imaging before sacrifice showed that high–surface PODXL cells presented strongly reduced PT size compared to low–surface PODXL cells (low surface, *n* = 9; high surface, *n* = 5; Fig. 7M). Macroscopic observation and weighing prostates after sacrifice allowed detection of additional small PTs in low–surface PODXL cells, indicating lower PT weight in high–surface PODXL cell tumors (low surface, *n* = 9; high surface, *n* = 7; Fig. 7N). There were differences in tumor morphology between high– and low–surface PODXL conditions (Fig. 7O). High–surface PODXL tumors displayed a less cohesive growth pattern with some preservation of acinar arrangement. These were much less circumscribed with infiltrative edges and a propensity to involve adjacent tissues, such as adipose tissue or seminal vesicle (four of five cases) and lymphovascular spaces (three of five cases). The low–surface PODXL group was solid in their growth pattern, although circumscribed with a rounded contour, and infiltration of adjacent tissues and lymphovascular spaces was only observed in one of six of the cases. Much of the low–surface PODXL tumor mass was necrotic with occasional islands of viable tumor seen adjacent to central capillaries.

Mirroring the infiltrative edges observed in high–surface PODXL PTs, this condition presented with a 100% metastasis rate, compared to a ~55% metastasis rate from low–surface PODXL cells (high surface PODXL, seven of seven mice; low surface PODXL, five of nine; *P* = 0.0417 using chi-square analysis; Fig. 7P). Moreover, high–surface PODXL cells displayed a ~1.8-fold increase in the number of organs presenting with macrometastasis per mouse with PT (Fig. 7Q), without altered tissue tropism (Fig. 7R). These data confirm an in vivo contribution of PODXL to metastasis with high surface PODXL levels representing a context that drives frequent metastasis.

### PODXL-GAL3 expression stratifies poor clinical outcome

In clinical samples, a high cortex-to-cytoplasm ratio for PODXL localization is a superior predictor of outcome than PODXL expression alone (*11*, *23*–*28*). Our studies mirror this, with only PODXL at the cell surface promoting invasion in vitro and high-level metastasis in vivo; we identify GAL3 as a key regulator of surface PODXL levels. We examined whether combining GAL3 expression with PODXL from tumors would allow stratification of patients with poor outcome.

**Fig. 8.**
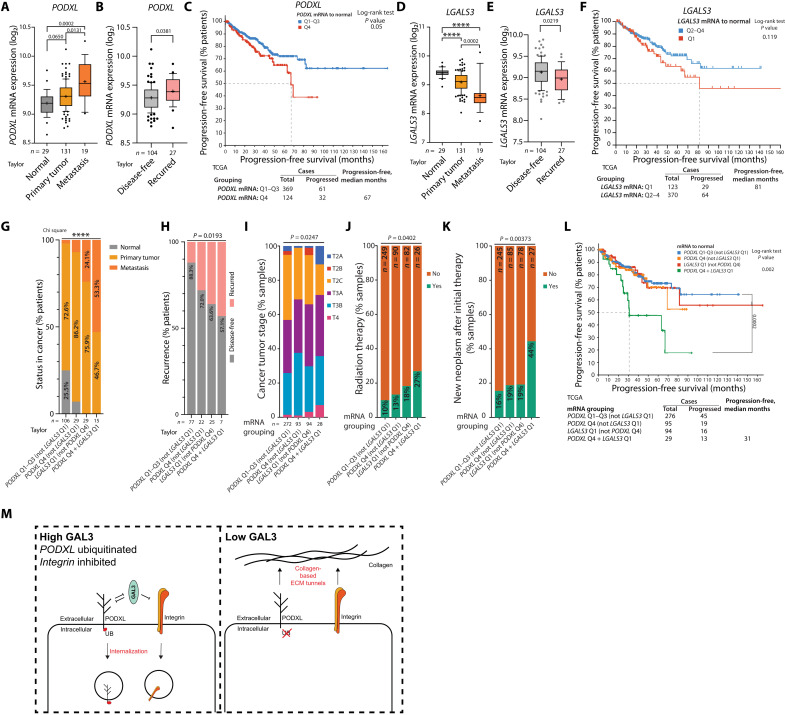
PODXL-GAL3 expression levels identify metastatic, treatment-resistant, and poor outcome tumors. (**A** and **B**) *PODXL* mRNA expression in (A) normal prostate, PT, and metastasis or (B) PTs in disease-free or recurred patients. (**C**) Progression-free survival (% patients, over months) in patient cohorts of high (Q4) versus not high (Q1 to Q3) *PODXL* mRNA expression. (**D** and **E**) *LGALS3* mRNA expression in (D) normal prostate, PT, and metastasis or (E) PTs in disease-free or recurred patients. (**F**) Progression-free survival (% patients, over months) in patient cohorts of low (Q1) versus not low (Q2 to Q4) *LGALS3* mRNA expression. (**G** to **L**) Clinical parameters in patient cohorts for combinations of high PODXL (Q4) and low *LGALS3* (Q1). (G) Sample type (normal, PT, metastasis; % patients). (H) Disease-free versus recurred status (% patients). (I) Tumor stage (% samples). (J). Radiation therapy status (% samples). (K) New neoplasm after initial therapy status (% samples). (L) Progression-free survival (% patients, over months). (**M**) Model, PODXL-GAL3 interplay controlling invasion. Box-and-whisker graphs (A, B, D, and E): 10th to 90th percentile; +, mean; dots, outliers; midline, median; boundaries, quartiles. Patient numbers presented in the figure. Statistical analyses, Kruskal-Wallis test (A and D), Welch’s *t* test (B and E), log-rank *t* test (C, F, and L), or chi-square test (G, H, and J to L). *P* values: stated, and ****P ≤ 0.0001. Data, Taylor (GSE21032) or TCGA (PRAD cohort).

*PODXL* mRNA levels were largely unchanged between normal prostate and PT tissue but were strongly elevated in metastatic samples and associated with disease recurrence across multiple patient cohorts (Fig. 8, A and B, and fig. S12, A to D). Comparison of patients based on quartiles (Q1 to Q4) of expression revealed that the highest *PODXL* expression (*PODXL*^HI^; Q4) identified patients with faster disease progression, with the effect sitting below or at the significance cutoff depending on the cohort (Fig. 8C and fig. S12, E and F). *LGALS3* mRNA displayed the inverted pattern wherein expression was strongly decreased in metastases and associated with disease recurrence across all cohorts but variably altered between normal tissue and PT (Fig. 8, D and E, and fig. S12, G to L). The lowest *LGALS3* expression (*LGALS3*^LO^; Q1) identified patients with accelerated disease progression in two of three cohorts (Fig. 8F and fig. S12, M and N).

We examined whether grouping patients based on the combination of *PODXL*^HI^ and *LGALS3*^LO^ expression would identify prostate cancer patients with different clinical parameters. We divided patients into four groups: (i) *PODXL* not high, (ii) *PODXL*^HI^ but not *LGALS3*^LO^, (iii) *LGALS3*^LO^ but not *PODXL*^HI^, and (iv) combined *PODXL*^HI^ and *LGALS3*^LO^(Fig. 8, G to K). This resulted in a stepwise decrease in clinical favorability in outcome for patients, with the *PODXL*^HI^-*LGALS3*^LO^ grouping identifying patients with the poorest outlook. The *PODXL*^HI^-*LGALS3*^LO^ combination was not found in normal tissue samples but (i) was present most frequently in metastases (53% of samples; Fig. 8G), (ii) was found most frequently in tumors from patients with recurring disease (42.9% patients; compare to 17.7% recurrence when *PODXL* was not high and *LGALS3* was not low; Fig. 8H), (iii) was associated with higher tumor stage (Fig. 8I), and (iv) occurred in patients that had received radiation therapy (Fig. 8J). Furthermore, *PODXL*^HI^-*LGALS3*^LO^ patients were 2.3-fold more likely to develop a new neoplasm after initial therapy (44% of patients; compare to 16 to 19% in other groups; Fig. 8K). The combination of *PODXL*^HI^-*LGALS3*^LO^ selected for patients with significantly reduced progression-free survival (Fig. 8L), improving on stratification based on PODXL or LGALS3 expression alone (Fig. 8, C and F). These data indicate that the collective interplay between PODXL-LGALS3 could be used as a potential biomarker of poor outcome, highly metastatic disease in patients with prostate cancer.

## DISCUSSION

The bulky tumor-associated glycocalyx can promote tumor growth and metastasis by regulating cell-ECM signaling, yet some of these components are apical membrane–located molecules in normal, apical-basal polarized epithelia, raising the question of how they function at the cell-ECM interface. Using PODXL as a model for the duality of these proteins, our data indicate that an additional context is required to control the localization of these components to the ECM interface.

Although PODXL has been implicated in the aggressiveness of multiple cancer types (*11*–*17*), the cellular mechanisms by which PODXL confers this function have remained largely unclear. Interaction of PODXL with a number of cytoplasmic proteins such as Ezrin/EBP50 (*54*), dynamin-2 (*55*), gelsolin (*56*), cortactin (*57*), and ARHGEF7/Rac1 (*13*) suggested a function of PODXL in regulating cytoskeletal dynamics. Key work from the Roskelley and McNagny laboratories indicated that in breast cancer cells, PODXL drives an Ezrin-dependent collective migration and tumor-budding phenotype (*58*). This Ezrin dependence was further corroborated in breast cancer cells when studying the role of PODXL in tumor cell extravasation, which curiously reported the PODXL extracellular domain as dispensable for extravasation (*39*). This is in contrast to blocking antibodies to the PODXL extracellular domain showing a decrease in tumor burden and metastasis (*22*). In our system, we find that the entire cytoplasmic domain of PODXL is dispensable for invasive activity. This suggests that there are key differences in how PODXL is contributing to tumorigenesis in different cell types. In prostate cancer cells, at least, we identify that at the surface, the major function of PODXL resides in its extracellular domain, acting as a rheostat on the integrin-repressing activity of the glycocalyx component GAL3.

Our findings allow us to present a model of how this GAL3-PODXL complex controls integrin-based invasion (Fig. 8M). Expression of PODXL alone is insufficient to stimulate invasion and instead requires PODXL retention mechanisms at the ECM-interacting membrane domain. Even in polarized epithelia where PODXL is normally apically localized, a pool of PODXL is delivered to the basolateral domain before retrieval in a phosphorylation-dependent fashion (*7*, *59*). Experimental mimicking of these phosphorylation events in PODXL induces PODXL localization to the ECM-abutting membrane and drives invasion. We here identify that ubiquitination is antagonistic to such retention. In the glycocalyx, GAL3 normally acts to repress integrin-driven invasion by triggering integrin internalization. Retention of PODXL at the ECM-abutting membrane relieves this integrin inhibition where PODXL acts as a decoy receptor for GAL3, controlling extracellular GAL3 uptake. Concomitantly, GAL3 acts to trigger PODXL internalization. The balance of PODXL-GAL3 modifies the glycocalyx to tune the invasion capacity of cells. A PODXL^HI^-GAL3^LO^ combination therefore allows for high-level integrin-based invasion, which we demonstrate occurs along collagen-lined invasion conduit tunnels. We report that differential ubiquitination of PODXL is a key event during EMT to control invasion.

The identity of the ubiquitin ligase and proteases that regulate PODXL ubiquitin directly remains unclear. The E3 ligase Nedd4-2 has been reported to control PODXL complex stability by regulating the ubiquitination of the PODXL complex component Ezrin but not PODXL itself (*60*). Counterintuitive to this model, we detected Nedd4-2 association only with high–surface PODXL interactome. Similarly, we do not yet know how ubiquitination affects PODXL fate. The Rab35 guanosine triphosphatase has been proposed to regulate tethering of PODXL in intracellular vesicles to the cell surface to promote surface PODXL retention (*61*–*63*), but whether ubiquitination modulates Rab35 association is unknown. Once internalized, a pool of PODXL can be delivered to exosomes, and the correct level of PODXL in exosomes is essential for the ability of these extracellular vesicles to confer invasive behaviors onto recipient cells (*63*). The release of PODXL in exosomes in vivo can also prepare the premetastatic niche for cell arrival by controlling ECM organization (*63*). Notably, this PODXL-regulated phenotype transfer also involves modulation of integrin trafficking and consequent remodeling of ECM, mirroring our findings. Ubiquitination of PODXL may therefore be a mechanism to fine-tune cell-autonomous invasive activity versus paracrine signaling to the stroma via exosomes, regulating a permissive ECM organization for invasion and metastasis. Whether PODXL also plays a role in matrix secretion remains unknown.

A key finding from our studies is that although all cells expressed PODXL, only some of these were able to undergo invasion. We identify that this context depends on the surface delivery of PODXL for its ability to modulate GAL3. The identification of GAL3-PODXL as a mutually repressive ligand-receptor pair, the balance of whose interplay affects integrin signaling, was unexpected as GAL1 association with the glycocalyx promotes ECM-integrin signaling in glioblastoma (*3*) and PODXL represses surface levels of β1-integrin in ovarian cancer cells (*23*). Moreover, GAL3 has been reported as a positive regulator of tumorigenesis in other contexts (*64*). Our findings revealed, contrastingly and in prostate cancer at least, that GAL3 is an inhibitor of integrin-dependent invasion with PODXL acting as a decoy to release GAL3-mediated repression of integrins. Why GAL3 may have opposing functionality in different tumor types is not known but may relate to whether PODXL presentation on the surface is required for tumorigenesis in all contexts. Targeting of PODXL to extracellular vesicles is one mechanism to influence metastasis in a non–cell autonomous fashion (*63*). Unraveling these key differences will be important to understanding how inhibition of PODXL could be used for clinical intervention.

Snyder *et al.* (*22*) demonstrated the first proof of concept that systemic treatment with PODXL-blocking antibodies is both tolerated and can reduce both PT and metastasis of MDA-MB-231 in murine xenografts. The requirement for surface PODXL presentation for metastasis further supports PODXL as an excellent target for therapeutic intervention with such antibodies. The identification of an interplay with GAL3 controlling whether PODXL contributed to invasion is clinically important. This provides a molecular mechanism for why high surface-to-cytoplasm ratio of PODXL in tumors is a superior indicator of disease aggressiveness than PODXL levels alone (*11*, *23*–*28*), and we now add that examination of transcript levels of the GAL3-PODXL axis identifies prostate cancer patients with advanced, recurrent, and metastatic disease. Accordingly, restricting PODXL participation in the cell-ECM glycocalyx by promoting either PODXL ubiquitination or treatment with anti-PODXL antibodies (*21*, *22*, *65*, *66*) may present attractive mechanisms to attenuate metastasis.

## MATERIALS AND METHODS

### Plasmids and cell lines

We used pLKO.1 puromycin lentiviral vector for stable knockdown or pLENTI-Crispr v2 (*67*) lentiviral vector for stable knockout of target proteins. Target sequences are listed in table S2. GFP control and GFP-tagged PODXL constructs (isoform A WT, ∆PBM, FBM*, ∆PBM + FBM*, ∆PBM + Ezrin, 5N>Q, 4K>R, FKBP-4K>R, ∆IC, ∆EC, and isoform B WT) were generated by subcloning using fragment synthesis (GeneArt, Thermo Fisher Scientific) and/or direct mutagenesis (Q5 Site-Directed Mutagenesis Kit, NEB) and cloned into the retroviral expression vector pQCXIZ by either restriction digestion and ligation or by In-Fusion cloning (Takara). FRB-BFP control and FRB-Ub-BFP constructs were generated by fragment synthesis and cloned into the retroviral expression vector pQCXIH. TagRFP-T control and TagRFP-T-LGALS3 constructs (WT and R186S mutant, both *LGALS3* RNAi resistant) were inserted into a modified version of the lentiviral expression vector pLEX303. In all instances, GFP-PODXL WT and mutants are based on the PODXLa splice variant (NM_001018111.3), except for where indicated that GFP-PODXLb (NM_005397.4) is used.

Stable cell lines were made by stable lenti- or retroviral transductions. For lentivirus, we cotransfected human embryonic kidney (HEK) 293-FT cells with plasmids of interest with vesicular stomatitis virus glycoprotein (pMD2.G, Addgene, plasmid 12259) and psPAX2 (Addgene, plasmid 12260) packaging vectors into HEK293-FT cells using Lipofectamine 2000 (Thermo Fisher Scientific). Viral supernatants at days 2 and 3 after transfection were collected, centrifugated at 3500*g*, filtered using polyethersulfone (PES) 0.45-μm syringe filters (Starlab) to remove cell debris, and concentrated using Lenti-X concentrator (Clontech) following the manufacturer’s protocol. Recipient cells were transduced with lentivirus for 72 hours before either selection with suitable antibiotic or fluorescence-activated cell sorting (FACS) for fluorescent protein using BD Fusion Sorter, where appropriate. For retroviral transfections, 293-GPG packaging cells were transfected with retroviral plasmids using Lipofectamine 2000, and viral supernatants were collected daily at days 5 to 7 after transfection and separated from cell debris as described above. Recipient cells were incubated with viral supernatants for 24 hours at 32°C with polybrene (10 μg/ml; Millipore), then the medium was changed, and cells were incubated for 48 hours at 37°C, before selection in appropriate antibiotic-containing medium and/or cell sorting for fluorescent proteins. Antibiotic concentrations used were as follows: blasticidin (5 μg/ml; InvivoGen), hygromycin (200 μg/ml; Merck), puromycin (2 μg/ml), G418 (300 μg/ml), or Zeocin (100 μg/ml; the last three are from Thermo Fisher Scientific).

### Cell culture

Cell lines were cultured as follows: PC3 [American Type Culture Collection (ATCC)] cells were cultured in RPMI 1640 (#31870) completed with 10% fetal bovine serum (FBS), 2 mM l-glutamine, and 1% penicillin/streptomycin. RWPE-1 cells (ATCC) were cultured in keratinocyte serum-free medium with bovine pituitary extract (50 μg/ml) and epidermal growth factor (5 ng/ml). MDA-MB-231 cells (ATCC) were cultured in Dulbecco’s modified Eagle’s medium (DMEM; #21969) supplemented with 10% FBS, 2 mM l-glutamine, and 1% penicillin/streptomycin. HEK293-FT cells (Thermo Fisher Scientific) were cultured in DMEM with 10% FBS, 2 mM l-glutamine, and 0.1 mM nonessential amino acids. 293-GPG cells (K. Mostov, University of California, San Francisco) (*68*) were cultured in DMEM with 10% heat-inactivated FBS, 2 mM l-glutamine, 25 mM Hepes, tetracycline (1 μg/ml; Sigma-Aldrich, T-7660), puromycin (2 μg/ml; Sigma-Aldrich, P-7255), and G418 (0.3 mg/ml). Unless specified annotated, all reagents were from Thermo Fisher Scientific. For SILAC-based analysis of PODXL interactome and total proteome, cells were metabolically labeled using SILAC DMEM (Life Technologies) supplemented with 10% 10-kDa dialyzed FBS (PAA), 1% glutamine, and 1% penicillin/streptomycin. SILAC medium used for the “medium”-labeled cells contained ^13^C_6_ l-arginine (84 mg/liter) and D4 l-lysine (175 mg/liter) and that for the “heavy”-labeled cells contained ^13^C_6_^15^N_4_ l-arginine (84 mg/liter) and ^13^C_6_^15^N^2^ l-lysine (175 mg/liter) (Cambridge Isotope Laboratories). Experiments were conducted when the incorporation rate of labeled amino acids was superior to 95%. All cells were routinely checked for mycoplasma contamination and authenticated using short tandem repeat (STR) profiling.

### 3D culture

3D culture of PC3, RWPE1, and MDA-MB-231 was performed as described in previous studies (*37*). Briefly, 96-well ImageLock plates (Essen Biosciences) or 96-well black/clear-bottom plates (Greiner, SLS) were precoated with 10 μl of Matrigel per well (BD Biosciences) for 15 min at 37°C. Single-cell suspensions (100 μl) at low density (1.5 × 10^4^ cells/ml) in appropriate medium containing Matrigel at 2% (PC3 and RWPE-1) or 4% (MDA-MB-231) were seeded on top of precoated wells. For 3D immunoblotting assays, six-well plates were precoated with 180 μl of Matrigel (BD Biosciences) per well for 20 min at 37°C. Single-cell suspensions (1.6 ml) at low-medium density (12.5 × 10^4^ cells/ml) in appropriate medium containing 2% Matrigel were seeded on top of precoated wells and harvested after 2 to 3 days. For treatment experiments, drugs, blocking antibodies, or the appropriate controls were added from the time of plating, unless otherwise specified. Drugs were added as follows: dimerization experiments, 200 μM A/C heterodimerizer (Takara) or ethanol control; for ReGal3 (R. Jacob, Philipps-Universität Marburg), 1.5 μM ReGal3 was added to cells; for β1-integrin inhibition, AIIB2 blocking antibody (8 μg/ml; DSHB) was added. Unless otherwise specified, three to four replicate wells were included per experiment as technical replicates, and experiments were repeated in at least three independent occasions.

### Live 3D cyst imaging and analysis

3D cultures were prepared on 96-well ImageLock plates (Essen Biosciences) as described in the “3D culture” section. Plates were then imaged at ×10 magnification hourly over 4 days using an IncuCyte ZOOM or IncuCyte S3 (Essen BioScience) with IncuCyte ZOOM Live Cell Analysis System Software 2018A or 2021A, respectively. Outlines of imaged objects were detected and generated using IncuCyte software, and then we applied a custom macro in ImageJ v1.51n to generate composite overlays of objects from 12 consecutive-hour time frames, rainbow color-coded by time. CellProfiler v3.1.9 (*69*) was used to obtain morphological features from resultant objects, and a Fast Gentle Boosting classifier was used to train and identify different behaviors (round, spread, and tunnel forming) in CellProfiler Analyst v2.2.1 (*70*). We then used custom pipelines using KNIME Data Analytics Platform v4.0.2 to combine data from multiple experiments, normalize to controls, calculate the *z* score, and perform statistical analysis using the Cochran-Mantel-Haenszel test and Bonferroni-adjusted to compare the proportion of each classification to control. Heatmaps of normalized data and *P* values were created using GraphPad Prism 9. Emphasized-track images were performed with ImageJ v1.51n by using “Remove Outliers, radius=25 threshold=2 function,” “Invert LUT,” and “Remove NaNs” and applying shadow functions. To analyze the speed of invasion of cells within the different classifications (round, spread, and tunnel forming), CellProfiler v3.1.9 was used to identify and mask outlines from objects within each behavior, and then ImageJ v1.51n was used to measure the movement of objects that appeared consistently in at least 12 consecutively outlined images, tracking the nucleus position.

### Immunofluorescence in 2D cell/3D cysts, imaging, and analysis

3D cultures were prepared on 96-well black/clear-bottom plates (Greiner, SLS) as described in the “3D culture” section. For 2D, cells were plated on the same plates 48 hours before fixation. In both cases, cells were gently washed in phosphate-buffered saline (PBS) before fixation in 4% paraformaldehyde (Thermo Fisher Scientific) for 10 min and then washed twice in PBS. Samples were blocked in PFS (0.7% fish skin gelatin/0.025% saponin/PBS) for 1 hour at room temperature with gentle shaking, followed by the addition of Alexa Fluor primary antibodies diluted 1:100 (unless otherwise specified) in PFS overnight (O.N.) at 4°C. Three washes in PBS were followed by the addition of Alexa Fluor secondary antibodies (1:200), Alexa Fluor phalloidin (1:200), or Hoechst (1:1000) (all from Thermo Fisher Scientific) diluted in PFS for 1 hour at room temperature and three washes in PBS. For staining of surface proteins, primary antibody diluted in fresh complete medium was added to live cells for 30 min at 4°C to label the surface proteins and to prevent internalization. Then, cysts were gently washed in PBS, fixed, and blocked as described above before appropriate secondary antibodies or dyes were added for 1 hour at room temperature, followed by three washes in PBS. Plates were imaged using either a Zeiss 880 laser scanning microscope with Airyscan or an Opera Phenix high-content analysis system (PerkinElmer).

For surface/total PODXL quantification, images were processed using Harmony imaging analysis software (PerkinElmer, Harmony 4.9). Briefly, cells expressing GFP-tagged variants of PODXL were stained with phalloidin to mark the cortex, Hoechst 34580 to mark the nuclei, and surface GFP to detect the intensity of cortical PODXL. A combination of these stainings was used to create masks that allow generation of cell regions (cortical, cytoplasmic, and nuclear), followed by calculation of the mean intensity in each region. For quantification of endogenous surface PODXL, fluorophore signal intensity of surface PODXL and F-actin were detected using ImageJ v1.51n.

### Wound healing migration and invasion assay

ImageLock 96-well plates (Essen Biosciences) were used for wound healing assays. For invasion, plates were precoated with 20 μl of 1% Matrigel (Corning) diluted in medium O.N. at 37°C. Cells were resuspended in complete medium at a density of 7 × 10^5^ cells/ml, and 100 μl was added per well. After 4 hours of incubation at 37°C, cells forming a monolayer were wounded using a wound-making tool (Essen Biosciences), washed twice with PBS to remove debris, and overlaid with either 100 μl of medium (for migration assays) or 50 μl of 25% Matrigel in complete medium, followed by the addition of 100 μl of complete media after incubation at 37°C for 1 hour to allow Matrigel polymerization (for invasion assays). Wound closure was imaged every hour for 4 days using IncuCyte ZOOM or IncuCyte S3 (Essen BioScience). Analysis of relative wound density (RWD) was performed using IncuCyte software. Results were normalized to control using Microsoft Excel 16.53 and presented as RWD at each time point (using R v3.6.2) or, unless otherwise specified, at the time point at which the average RWD of control samples reached 50% of confluence (*T*_max_^1/2^) using GraphPad Prism 9.

### Cell viability assays

2D and 3D cell viability assays were performed using CellTiter-Glo 3D Cell Viability Assay (Promega) as per the manufacturer’s instructions. Briefly, cells were resuspended at low density (1.5 × 10^4^ cells/ml) either in complete medium for 2D assays or complete media containing 2% of Matrigel for 3D assays. Cell suspension (100 μl) was added per well on black/clear-bottom 96-well plates (Greiner) either uncoated or precoated with 10 μl of Matrigel for 2D and 3D assays, respectively. Equilibrated CellTiter-Glo reagent (100 μl) was added to each well at days 0, 1, 2, 3, or 5 after plating; mixed; and prepared for luminescence detection using a Tecan Safire 2 and SparkControl v2.3 software. Statistical analysis was performed using Microsoft Excel 16.57 and GraphPad Prism 9.

### Flow cytometry and FACS

For cortical protein localization, cells were washed twice in ice-cold PBS, detached using 2 mM EDTA in PBS for 15 min, and resuspended in PBS + 2% FBS. Cells were counted, and 3 × 10^5^ cells per sample were blocked for 30 min at 4°C with primary antibodies (5 μg per million cells, unless otherwise specified; table S3) and then washed and stained with a corresponding secondary conjugated antibody (1:200; Alexa Fluor, Thermo Fisher Scientific) for 30 min at 4°C. After washing, samples were processed with BD Fortessa Z6102 (BD FACSDiva software, v8.0.1) and then analyzed with FlowJo software (version 10.1r5) and GraphPad Prism 9. For sorting of cell lines stably expressing fluorescent-tagged proteins, cells were trypsin-detached, resuspended in PBS + 2% FBS, strained through a 40-μm EASTstrainer (Greiner), and processed using a BD Fusion Sorter (BD FACSDiva software, v8.0.1).

### Immunoblotting

When samples were obtained from cell lysates harvested on plastic (2D), cells at ~80% confluency were washed with cold PBS. Ice-cold lysis buffer [50 mM tris-HCl (pH 7.4), 150 mM NaCl, 0.5 mM MgCl_2_, 0.2 mM EGTA, and 1% Triton X-100] with cOmplete protease inhibitor cocktail and PhosSTOP tablets (Roche) was added for 15 min. Cells were scraped and passed through a 27G x 1/2'' -gauge needle before centrifugation at 14,000*g* at 4°C for 15 min, and then the supernatant was collected. A MicroBCA Protein Assay Reagent kit (Pierce) was used for protein quantification. To obtain cell lysates of 3D samples, a similar procedure was followed with the particularity that after the centrifugation step, only the top phase of the supernatant was collected, not collecting the semiopaque layer between the pellet and supernatant, rich in ECM components. To calculate protein concentration, 10 μl of each sample was immunoblotted and probed for glyceraldehyde-3-phosphate dehydrogenase (GAPDH).

To identify proteins from extracellular media, cells were seeded in 100-mm plastic plates and permitted growth until they were in exponential phase (~80% confluent). Complete media was removed, followed by three washes in PBS to remove most traces of FBS, and incubated on 7 ml of serum-free media for 24 hours. Working at 4°C onward, the medium was collected and serially centrifuged at 300, 2000, and 10,000*g* for 10, 10, and 30 min, respectively, collecting the supernatant after each step. Supernatant pH was adjusted to a final pH of 5 by adding 10% of trifluoroacetic acid (TFA). Strataclean beads (70 μl; Agilent Technologies) were added (10 μl of beads per milliliter of media), followed by an incubation step for 1 hour in a rotor wheel. Beads bound to supernatant proteins were collected by centrifugation at 872*g* for 1 min, supernatant was discarded, and 35 μl of loading buffer was added to the beads and then boiled at 95°C for 5 min.

In all instances, SDS–polyacrylamide gel electrophoresis protein separation in Bolt 4 to 12% bis-tris plus gels (Thermo Fisher Scientific) was followed by protein transference to polyvinylidene difluoride (PVDF) membranes using an iBlot 2 transfer system (Thermo Fisher Scientific). Membranes were incubated for 45 min in Rockland blocking buffer (Rockland), followed by primary antibody (listed in table S3) binding O.N. at 4°C (1:1000 unless stated otherwise). After three washes in TBST (tris-buffered saline with 0.1% Tween 20 detergent), appropriate secondary antibodies were added for 1 hour to membranes and then washed again in TBST and imaged using the Odyssey Imaging System (LI-COR Biosciences) or ChemiDoc Imager (Bio-Rad). Band quantification was performed using Image Studio Software 6.0 (LI-COR Biosciences) or Image Lab 6.1 (Bio-Rad), respectively. Graphs were generated on GraphPad Prism 9. GAPDH was used as a loading control for each immunoblot, and a representative image for each sample set is shown where appropriate. Uncropped membranes are presented in fig. S13.

### Co-IP, GFP-Trap, and UB-Trap

Cell lysates were obtained as described above, using lysis buffer as recommended by the manufacturers [for coimmunoprecipitation (Co-IP) and GFP-Trap, 10 mM tris-Cl (pH 7.5), 150 mM NaCl, and 0.5% NP-40 with 1× cOmplete protease inhibitor cocktail and PhosSTOP tablets (Roche); for ubiquitin-Trap (UB-Trap), BlastR Lysis Buffer (# BLST01, Cytoskeleton) with 1× protease inhibitor cocktail (PIC02, Cytoskeleton) and 1× deubiquitination/small ubiquitin-like modifier (SUMOylation) inhibitor (*N*-ethylmaleimide and *N*,*N*,*N′*,*N′*-tetrakis(2-pyridylmethyl)ethylenediamine, NEM09BB, Cytoskeleton)]. To identify relevant PODXL protein-protein interactions by Co-IP, 1 mg of cell lysate was immunoprecipitated with 2 μg of anti-PODXL antibody (3D3, Thermo Fisher Scientific) O.N. at 4°C with rotation. Protein complexes were identified by adding anti-mouse immunoglobulin G (IgG) agarose (and mouse IgG agarose as a control; #A6531 and #A0919, respectively, both from Sigma-Aldrich) for 1 hour at 4°C in rotation, before three washes in lysis buffer. Immunocomplexes were unbound from beads by resuspension in loading buffer with a sample-reducing agent and heated in a heat block for 10 min at 100°C. Protein separation and transfer to PVDF membranes were as described above. For lysates of cells expressing GFP-tagged proteins, immunoprecipitation was performed using the GFP-Trap Kit (Chromotek) as per the manufacturer’s instructions. For ubiquitinated protein detection, ubiquitination enrichment immunoprecipitation was performed using a Signal-Seeker Ubiquitination Detection kit (Cytoskeleton) as per the manufacturer’s instructions. For FKBP-FRB dimerization system experiments, cells were incubated in media containing 200 μM A/C heterodimerizer (Takara) or ethanol control for 4 hours before lysis. In all cases, subsequent immunoblotting was performed as described in the immunoblotting method.

### Reverse transcription quantitative polymerase chain reaction

For detection of mRNA transcriptional levels of *PODXL* among cell lines, reverse transcription quantitative polymerase chain reaction (RT-qPCR) was used. RNA was extracted using an RNeasy kit (Qiagen), followed by RT using a High-Capacity cDNA (complementary DNA) RT kit (Thermo Fisher Scientific). RT-qPCR was performed using TaqMan Master Mix and TaqMan primers: PODXL: Hs01574644_m1; GAPDH: Hs02758991_g1 (Thermo Fisher Scientific), per the manufacturer’s instructions. Expression was normalized to GAPDH levels between cells. Three independent samplings of RNA from a single biological condition were used, with one technical replicate per experimental sampling.

### RNA sequencing

RNA-seq was performed as described in previous studies (*71*). Briefly, RNA was extracted as described in the “Reverse transcription quantitative polymerase chain reaction” section, including a deoxyribonuclease (DNase) digestion step using a ribonuclease-free DNase set (Qiagen). Quality control of all RNA samples was performed using a 2200 Tapestation and High-sensitivity RNA screentape (Agilent) to measure the quality of the purified RNA, and only those with RIN values of >7.9 were processed, with 1 μg of total RNA used as initial input. Libraries were then prepared using the manufacturer’s standard procedures (Illumina TruSeq stranded mRNA). Libraries were then sequenced on an Illumina NextSeq 500 instrument, on a high-output 75-cycle run with paired-end 36–base pair read length.

For data analysis, quality checks and trimming on the raw RNA-seq data files were done using FastQC version 0.11.8, FastP version 0.20, and FastQ Screen version 0.13.0. RNA-seq paired-end reads were aligned to the GRCm38 version of the human genome and annotation using HiSat2 version 2.1.0. Expression levels were determined and statistically analyzed by a combination of HTSeq version 0.9.1 and the R environment version 3.6.1, using packages from the Bioconductor data analysis suite and differential gene expression analysis based on the negative binomial distribution using the DESeq2 package version 1.22.2. Pathway analysis was preformed using MetaCore from Clarivate Analytics (https://portal.genego.com/).

### MS: Sample preparation

For evaluation of SILAC labeling, 20 µg of total lysates from cells grown in SILAC medium was used for in-solution protein digestion. Proteins were reduced using 1 mM dithiothreitol (Sigma-Aldrich) for 1 hour at room temperature and alkylated using 5.5 mM iodoacetamide (Sigma-Aldrich) for 45 min at room temperature protected from light. A predigestion step was performed with Lys-C (Alpha Laboratories) for 3 hours at room temperature. Then, peptides were diluted fourfold with ABC buffer [50 mM ammonium bicarbonate (pH 8)] and digested with trypsin MS grade (Promega) O.N. at room temperature. The next day, samples were acidified to pH <4.0 using 1% TFA (Sigma-Aldrich). Peptides were desalted and concentrated using C18 Stage Tips.

For the interactome analyses of PODXL, SILAC mixtures were loaded onto 4 to 12% SDS–polyacrylamide gels for electrophoretic separation of proteins. Gel was stained, and each gel lane was divided into 8 to 10 slices. Proteins were digested into peptides using an in-gel digestion method (*72*).

For the total proteome analysis of PODXL, 200 µg of protein (mixture containing 100 µg of protein derived from each SILAC condition) was lysed in SDS buffer [4% SDS, 100 mM dithiothreitol, 100 mM tris-HCl (pH 7.6)]. Proteins were digested into peptides using the filter-aided sample preparation method (*73*). Subsequently, 50 µg of peptides was fractionated using strong anion exchange (SAX) chromatography on Stage Tips.

### MS data acquisition

Purified tryptic peptides were injected on an EASY-nLC system coupled online to an LTQ-Orbitrap Pro Elite, through a nanoelectrospray ion source (Thermo Fisher Scientific). An active background ion reduction device (SmartSource Solutions LLC) was used to decrease air contaminants. Peptides were separated on a 20-cm fused silica emitter (New Objective) packed in-house with reverse-phase Reprosil Pur Basic 1.9 mm (Dr. Maisch GmbH). The emitter was heated to 35°C using a column oven (Sonation). The peptides were eluted with a two-step gradient at a flow rate of 200 nl/min, with buffer A (0.1% formic acid) and buffer B (80% acetonitrile and 0.1% formic acid). The gradient length for in-gel digestion bands was 150 min and for the SAX fractions was 255 min. MS data were acquired in a positive ion mode using data-dependent acquisition with XCalibur software (Thermo Fisher Scientific). The mass range acquired for the full MS scan was 300 to 1650 mass/charge ratio (*m*/*z*), with a resolution of 120,000 at 400 *m*/*z*. Only multiply charged ions from two to five charges were selected for fragmentation. For PODXL IP samples, higher-energy collision dissociation fragmentation was triggered for the 10 most intense ions, using a maximum injection time of 150 ms or a target value of 40,000 ions, at a resolution of 15,000. For PODXL proteome samples, collision-induced dissociation fragmentation was triggered for the 10 most intense ions, using a maximum injection time of 25 ms or a target value of 5000 ions. A dynamic exclusion window of 60 s was used for ions that had already been selected for MS/MS.

### MS data analysis

The MS .raw files were processed with the MaxQuant software version 1.5.0.36. The Andromeda search engine performed the search against the human UniProt database (release 2013, 88,847 entries). The multiplicity was set accordingly to the SILAC labels of each experiment, and remainder parameters included a minimal peptide length of seven amino acids, variable modifications acetyl (protein N-term) and oxidation (M), fixed modification carbamidomethyl (C), specificity for trypsin cleavage, and maximum of two missed cleavages allowed. For the search of the parent mass and fragment ions, an initial mass deviation of 4.5 and 20 parts per million, respectively, was required. The requantify parameter was enabled, and match between runs was used between similar fractions. The false discovery rate at the protein and peptide level was set to 1%. Unique peptides with at least two ratio counts were used. The MaxQuant output table “ProteinGroups.txt” was uploaded in Perseus version 1.5.2.11 for downstream analysis. Datasets were filtered to remove contaminants, reverse peptides that match a decoy database, and proteins only identified by site. Proteins with at least one unique peptide were used for analyses. Normalized SILAC ratios were log_2_-transformed, and intensities were log_10_-transformed. To organize PODXL-interacting proteins into a functional cluster, we used Cytoscape v3.9.0 with integrated STRING network analysis (0.7 confidence score).

### Internalization assay by capture ELISA

Protein internalization was measured as described in (*53*). Briefly, cells were surface-labeled with NHS-SS-biotin (0.13 mg/ml; Pierce) in PBS at 4°C for 30 min. Internalization was reestablished by placing cells in complete media in the presence of 0.6 mM primaquine to disrupt vesicle recycling for the indicated times. Cell were placed on ice, and biotin was removed from the cell surface by MesNa treatment. Following that, cells were lysed, and the quantity of biotinylated receptors was determined using capture ELISA. Antibodies used α5β1 (VC5, BD Pharmingen, 555651), TfnR (CD-71, BD Pharmingen, 555534), and GFP (Abcam, ab1218).

### Internalization assay by immunofluorescence

ReGal3 internalization was measured by immunofluorescence. Briefly, 1.5 μM ReGal3 was bound to the cell surface of PC3 cells in 2D culture for 15 min at 4°C. Internalization was reestablished by placing cells at 37°C for 0, 2, and 10 min. Cells were washed twice in PBS, then fixed in 4% PFA (Thermo Fisher Scientific) for 10 min, and washed in PBS. ReGal3 was detected by immunofluorescence using primary antibody anti-Gal3 following the procedure described in the “Immunofluorescence in 2D cell/3D cysts, imaging, and analysis” section.

### Cell attachment

Ninety-six-well black/clear-bottom plates (Greiner) were precoated with 20 μl of different ECMs [1:100 laminin (#23017015, Thermo FIsher Scientific), 1:100 or 10 μg/ml of fibronectin (#F1141, Sigma-Aldrich), 1:200 or 10 μg/ml of collagen I (354236, Corning), or 50% Matrigel (BD Biosciences) in PBS] and incubated O.N. at 37°C. Cells were detached by mild methods (2 mM EDTA in PBS for 15 min) and counted. Cells (1500) in 100 μl were added per precoated well and incubated at 37°C for 30, 60, 120, or 240 min, followed by a wash in prewarmed PBS, fixation, and staining, as previously described in the “Immunofluorescence in 2D cell/3D cysts, imaging, and analysis” section.

### Animal studies

Animal experiments were performed in compliance with the ethical regulations of U.K. Home Office Project License (P5EE22AEE), approved, and sanctioned by the welfare and ethical review board of the University of Glasgow under the Animal Scientific Procedures Act 1986 and the European Union directive 2010. CD-1 nude (CD1-*Foxn1^nu^*) male mice (6 weeks of age) were obtained from Charles River Laboratories (UK) and acclimatized for at least 7 days. Mice were kept in a barriered facility at 19° to 22°C and 45 to 65% humidity in 12-hour light/12-hour dark cycles with access to food and water ad libitum and environmental enrichment. A cohort of 12 mice (PC3 shRNA Scramble or PODXL) or 10 mice (PC3-high or PC3-low) were used per experiment. Surgery (under anesthesia and with analgesia) was performed to implant 2 × 10^6^ PC3 cells within the anterior prostate lobe of each mouse, followed by close monitoring of tumor development (including by palpation) and health conditions, until the end point of the experiment, 8 weeks after implantation. PTs were imaged and measured (volume) using a VevoLAB ultrasound equipment and VevoLAB 3.1.1 software. Metastases were analyzed by gross observation in the bladder, seminal vesicle, lymph nodes, epididymal fat, abdominal walls, mesenteric mass, pancreas, spleen, kidney, liver, body cavity, diaphragm, and lungs. Statistical analysis was performed using chi-square test (with Fisher’s exact test) in GraphPad Prism 9.

### H&E and IHC staining

All hematoxylin and eosin (H&E) and immunohistochemistry (IHC) staining was performed on 4-μm formalin-fixed paraffin-embedded (FFPE) sections, which had previously been maintained at 60°C for 2 hours. FFPE sections for Gal3 (87985, Cell Signaling Technology) IHC staining were dewaxed using an Agilent pretreatment module. Sections were then heated to 97°C for 20 min in a high-pH target retrieval solution (K8004, Agilent) for heat-induced antigen retrieval, washed in flex wash buffer (K8007, Agilent), and loaded onto an Agilent autostainer link48. Sections were blocked with peroxidase (S2023, Agilent) for 5 min and washed with flex wash buffer, and Gal3 was applied (1/600) for 35 min. Sections were washed with flex wash buffer, and rabbit envision secondary antibody (K4003, Agilent) was applied for 30 min. After another wash in flex wash buffer, liquid DAB (K3468, Agilent) was added for 10 min. The sections were washed in water and counterstained with hematoxylin z (RBA-4201-00A, CellPath).

FFPE sections for Podxl (ab15038, Abcam) were stained on a Leica Bond Rx autostainer undergoing on-board dewaxing (AR9222, Leica) and epitope retrieval using ER2 solution (AR9640, Leica) for 20 min at 95°C. Sections were washed with Leica wash buffer (AR9590, Leica) before peroxidase block was performed using an Intense R kit (DS9263, Leica). FFPE sections were rinsed with wash buffer, and Podxl antibody was applied for 30 min (1/1000; Abcam). Sections were rinsed with wash buffer, and rabbit envision secondary antibody was applied for 30 min. After a final wash in wash buffer, sections were visualized using DAB and counterstained with hematoxylin from an Intense R kit.

H&E staining was performed on a Leica autostainer (ST5020). Sections were dewaxed, applied with graded alcohols, and then stained with Haem Z (RBA-4201-00A, CellPath) for 13 min. Sections were washed in water, differentiated in 1% acid alcohol, washed, and the nuclei blu’d in Scotts tap water substitute. After washing with tap water, sections were placed in Putt’s eosin for 3 min. To complete the H&E and IHC staining, sections were washed in tap water, dehydrated through graded ethanol, and placed in xylene. The stained sections were coverslipped from xylene using DPX mountant (CellPath, UK). Stained FFPE sections were scanned at ×20 magnification using a Leica Aperio AT2 slide scanner and analyzed using Halo software (Indica Labs).

Following IHC, tumor areas were manually drawn and analyzed by area quantification using Halo software. Expressions were scored using histoscore [= Σ (1 × % area of weak positive staining) + (2 × % area of moderate positive staining) + (3 × % area of strong positive staining)]. All statistical analyses and graphs were made using GraphPad Prism 9.

### Analysis of cancer cell lines from CCLE

Data from a panel of breast and prostate cancer cell lines were obtained from the CCLE [Cancer Cell Line Encyclopedia, Broad Institute (*74*)] and analyzed using GraphPad Prism 9.

### Analysis of patients

Patient data were obtained from cBioportal.org and CANCERTOOL (*75*) and analyzed using cBioportal.org tools and GraphPad Prism 9. Datasets include Glinsky (*76*), Grasso (GSE35988), Lapointe (GSE3933), Taylor (GSE211032), The Cancer Genome Atlas (TCGA; obtained from cBioportal.org), Tomlins (GSE6099), and Varambally (GSE3325).

## Notes

View/request a protocol for this paper from *Bio-protocol*.
